# B Cell Intrinsic STING Signaling Is Not Required for Autoreactive Germinal Center Participation

**DOI:** 10.3389/fimmu.2021.782558

**Published:** 2021-12-06

**Authors:** Kenneth Green, Thomas R. Wittenborn, Cecilia Fahlquist-Hagert, Ewa Terczynska-Dyla, Nina van Campen, Lisbeth Jensen, Line Reinert, Rune Hartmann, Søren R. Paludan, Søren E. Degn

**Affiliations:** ^1^ Department of Biomedicine, Aarhus University, Aarhus, Denmark; ^2^ Department of Molecular Biology and Genetics, Aarhus University, Aarhus, Denmark; ^3^ Department of Biomedical Sciences, Radboud University Medical Center, Nijmegen, Netherlands

**Keywords:** B cells, germinal centers, autoreactivity, autoimmunity, cGAS, STING, IFNAR, TLR7

## Abstract

Germinal centers (GCs) are induced microanatomical structures wherein B cells undergo affinity maturation to improve the quality of the antibody response. Although GCs are crucial to appropriate humoral responses to infectious challenges and vaccines, many questions remain about the molecular signals driving B cell participation in GC responses. The cyclic GMP-AMP synthase (cGAS)-stimulator of interferon genes (STING) pathway is an important mediator of type I interferon and proinflammatory cytokine responses during infection and cellular stress. Recent studies have reported important roles for STING in B cell responses, including an impact on GC B cells and downstream antibody responses, which could have great consequences for vaccine design and understanding STING-associated interferonopathies. GCs are also involved in untoward reactions to autoantigens in a plethora of autoimmune disorders, and it is generally thought that these responses coopt the mechanisms used in foreign antigen-directed GCs. Here, we set out to investigate the importance of the cGAS-STING pathway in autoreactive B cell responses. In a direct competition scenario in a murine mixed bone marrow chimera model of autoreactive GCs, we find that B cell intrinsic deficiency of cGAS, STING, or the type I interferon receptor IFNAR, does not impair GC participation, whereas Toll-like receptor (TLR)-7 deficiency mediates a near-complete block. Our findings suggest that physiological B cell responses are strictly sustained by signals linked to BCR-mediated endocytosis. This wiring of B cell signals may enable appropriate antibody responses, while at the same time restricting aberrant antibody responses during infections and in autoimmune or autoinflammatory settings.

## Introduction

Antibodies are an important constituent of the adaptive immune response. During an ongoing response, antibodies are refined through a process called affinity maturation to attain high specificity and affinity against their cognate antigen. This process takes place in distinct anatomical locations inside the follicles of lymphoid tissues, termed germinal centers (GCs). Here, B cells activated by their cognate antigen undergo iterative cycles of clonal expansion with somatic hypermutation and selection towards affinity for antigen, to yield progressively higher affinity antibodies (1, 2). Ultimately, the selected B cell clones differentiate into either antibody producing plasma cells (PCs) or memory B cells. This process contributes greatly to the efficiency, plasticity and versatility of the humoral immune response against foreign antigens (3). Unfortunately, the pathogenesis of multiple autoimmune disorders is also influenced by such GC reactions, which contribute to the initiation and propagation of disease (4, 5). A prominent example is Systemic Lupus Erythematosus (SLE) (6), wherein GC reactions foster a phenomenon termed B cell epitope spreading (7, 8). In epitope spreading, the B cell pool expands and diversifies their antibody repertoire, which ultimately results in a diversification of the antigenic determinants targeted by the immune system. While this process is thought to contribute to robust protection against pathogens, it also plays a central role in propagating autoreactivity (9, 10). It has been noted that years before patients present with SLE, they evolve autoantibodies targeting an increasing breadth of autoantigens, and this is thought to drive the pathogenic process (11).

Despite the vital importance of robust B cell participation in GC reactions, and the dangers of untoward GC responses, many questions remain about the essential components and intracellular mechanisms involved. In the case of autoreactive GC responses, it has been found that B cell intrinsic signals driven by endogenous Toll-like receptor (TLR)-7 ligands are a main determinant of GC inclusion (12–14). The role of TLR9 has been debated, but the current consensus is that whereas TLR7 is pathogenic, TLR9 is protective (15). It has been suggested that autoreactivity first focuses on self-components which carry TLR ligands, because such dual-specific antigens will uniquely be able to activate B cells independently of T cells, with subsequent T–B interactions activating autoreactive T cells, resulting in chronic autoimmunity (16).

Whereas the B cell intrinsic role of TLRs has been the subject of intense scrutiny, much less is known about the multitude of cytosolic nucleic acid sensing pattern recognition receptors (PRRs), which may trigger signaling cascades with a vast variety of downstream responses (17). The cyclic GMP-AMP synthase (cGAS)-stimulator of interferon genes (STING) pathway is important in the recognition of cytosolic double-stranded (ds)DNA structures. cGAS ligands have various sources and can originate from pathogens, like bacteria and viruses, or from self-DNA, cytosolically introduced by, e.g., genome destabilization, mitochondrial damage or phagocytosis of dead neighbor cells (18–20). The pathway is central in diseases like Aicardi–Goutières syndrome (AGS) and STING-associated vasculopathy with onset in infancy (SAVI), but has also been implicated in SLE (21–24).

One of the main downstream targets of STING activation is the induction of type I interferons (IFNs). Type I IFNs, produced auto- or paracrinely, in turn act on the Interferon-α/β Receptor, IFNAR, a heterodimer receptor complex of IFNAR1:IFNAR2. IFNAR signals *via* the JAK-STAT pathway, through JAK1/TYK2 and STAT1/2, to induce expression of multiple IFN-stimulated genes (ISGs). B cells express IFNAR, and type I interferons have previously been reported to modulate B cell physiology at several levels, including selection of naïve B cells into responses and a direct effect on GC B cells (25). Of interest in relation to the previously noted role of TLR signaling in B cells, it was reported that an IFN-β feedback loop regulates the B cell intrinsic expression of TLR7, hence determining the sensitivity of B cells to TLR7 mediated signals (26). Surprisingly, however, several recent studies found that knock-out of IFNAR did not impact GC B cell responses in *in vivo* models of autoreactivity, whereas IFN-γ was a critical component of the response (27–29).

While the roles of STING in stromal cells and immune cells of the myeloid lineage are well-established, much less is known about its functions in lymphocytes. However, several papers have reported that STING signaling in T cells can induce apoptosis (30, 31). In addition to accelerating cell death, STING activation in T lymphocytes could also prevent cell proliferation (32). This finding was extended by the observation that patients carrying constitutive active mutations in STING harbor a reduced number of T memory cells and display impaired T cell proliferation. Similar roles for STING in B cells could be envisioned to limit GC responses. Although the B cell intrinsic roles of STING signaling are still unclear, STING was recently suggested to be coupled to the B cell receptor (BCR) signal transduction in B cell activation (33–35).

DNA reactive B cells are a hallmark of lupus (36). Because the cGAS-STING pathway has been implicated in autoinflammatory and autoimmune conditions, we chose to investigate the role of STING in a murine mixed bone marrow (BM) chimera model of autoimmunity resembling SLE, with a prominent type I interferon signature. Notably, this model is based on an autoreactive driver, the 564Igi knock-in clone, which was originally identified based on its high affinity for DNA (12). As demonstrated recently, in the mixed chimera model, an epitope spreading process occurs, broadening the autoantigenic targets from this original focus on DNA to a plethora of additional autoantigenic targets (37). Hence, this model should be well suited to interrogate autoimmune processes stemming from initial recognition of nucleic acids and manifesting through a broadening autoreactivity, as also observed in SLE patients (11).

## Materials and Methods

### Mice

C57BL6/JRj (B6; CD45.2) were purchased from Janvier, congenic B6.CD45.1 (B6.SJL-Ptprc^a^ Pepc^b^/BoyJ, JAX stock no. 002014), Photo-Activatable (PA)-GFP transgenic mice (38) (B6.Cg-Ptprc^a^ Tg(UBC-PA-GFP)1Mnz/J, JAX stock no. 022486), and TLR7 knockouts (39) (B6.129S1-Tlr7^tm1Flv^/J, JAX stock no. 008380) were obtained from the Jackson Laboratories. 564Igi mice (12) (B6.Cg-Igh^tm1(Igh564)Tik^Igk^tm1(Igk564)Tik^/J) were kindly made available by Theresa Imanishi-Kari and provided by Michael C. Carroll, Boston Children’s Hospital. IFNAR knockouts (B6(Cg)-Ifnar1^tm1.2Ees^/J, JAX stock no. 028288), STING-Gt (C57BL/6J-Sting1^gt^/J, JAX stock no. 017537), and *Mb21d1* (cGAS) knockout mice (40) (B6(C)-*Cgas^tm1d(EUCOMM)Hmgu^*/J, JAX stock no. 026554) were obtained at Jackson Laboratories and bred at Taconic Denmark. Mice were housed in the Animal Facility at Department of Biomedicine, Aarhus University, Denmark, under specific pathogen-free (SPF) conditions, on a 12-hour light/dark cycle with standard chow and water *ad libitum*. Donors were 6-9, 7-11, 6.5-14 or 9 weeks old, and recipients were 11, 6-8, 9 or 7 weeks old in the STING-Gt, TLR7, cGAS and IFNAR setups, respectively, and both male and female mice were used.

### Ethics Statement

All animal experiments were conducted in accordance with the guidelines of the European Community and were approved by the Danish Animal Experiments Inspectorate (protocol number 2017-15-0201-01348).

### Antibodies and Staining Reagents

Commercial antibodies and staining reagents originated from BioLegend: CD45.1-FITC, CD45.2-APC, CD38-PE-Cy7, CD4-PerCP, CD8a-PerCP-Cy5.5, IFNAR1 biotin, and streptavidin conjugated to BV786; from BD Biosciences: CD16/CD32, CD138-BV650, B220-PacBlue, CD95 (APO-1/Fas)-PE; from ThermoFisher Scientific: GL7-A488, Ki67-efluor660 and viability dye fixable live/dead stain eFlour780. The 9D11 hybridoma expressing anti-idiotypic monoclonal IgG1 antibody, clone 9D11 (41), was kindly provided by Elisabeth Alicot, Boston Children’s Hospital, and was conjugated with iFluor647 succinimidyl ester (AAT Bioquest) in-house.

### Mixed Bone Marrow Chimeras

The day before the reconstitution, recipient mice were irradiated with 9 Gy in a MultiRad 350 (Faxitron), with settings of 350 kV, 11.4 mA, a Thoraeus filter [0.75 mm Tin (Sn), 0.25 mm Copper (Cu), and 1.5 mm Aluminum (Al)], and with a beam-distance of 37 cm. Irradiated recipients were kept on antibiotic water (either 1 mg sulfadiazine together with 0.2 mg trimethoprim per ml drinking water, or 0.25 mg amoxicillin per ml drinking water) to avoid any opportunistic infections. On the day of reconstitution, donor mice were anesthetized with 4% isoflurane in air and euthanized by cervical dislocation. *Femora, fibulae/tibiae, ossa coxae* and *humeri* were harvested, mechanically cleaned and rinsed in BM buffer [Phosphate-buffered saline (PBS) (137 mM NaCl, 2.7 mM KCl, 10 mM Na_2_HPO_4_ and 1.8 mM KH_2_PO_4_; pH 7.4), 2% heat-inactivated Fetal Bovine Serum (FBS), 1 mM Ethylenediaminetetraacetic acid (EDTA)]. The BM cells were released from the harvested bones by crushing in a mortar with ice cold BM buffer and the cell extract was then passed through a 70 μm cell strainer. The strained donor BM cells were counted in a Cellometer K2 cell counter (Nexcelom) following the manufacturer’s instructions. Cells were pelleted by centrifugation (200 g, 4°C, 10 min) and resuspended to get a total cell concentration of 1·10^8^ cells per ml. Donor cells of different origin were then mixed according to the needed proportions (*e.g.* 1/3 564, 1/3 CD45.1 and 1/3 CD45.2) as indicated in the figure legends of the individual setups.

The donor cell mixtures were used to reconstitute the myeloablated recipient mice by retroorbital injection of 200 μl (containing a total of 20·10^6^ cells) into each anesthetized recipient mouse. The anesthetization was done with an Isoflurane vaporizer, using 4% isoflurane (Attane Vet, ScanVet) for the induction phase, followed by 2-3% for maintenance. Anesthesia was verified throughout the procedure by testing the pedal withdrawal reflex upon pinching. The reconstituted recipient mice were placed on antibiotic water (acidified drinking water containing 1 mg of sulfadiazin and 0.2 mg of trimethoprim/ml, or ultrafiltered water containing 0.25 mg amoxicillin/ml) the following 14 days, and for the TLR7-KO, cGAS-KO and the second STING-Gt cohort additionally 14 days before euthanasia.

### Flow Cytometry

Six weeks after the reconstitution, the chimerism of the reconstituted recipient mice was evaluated by blood samples. Retroorbital blood samples were taken from each individual chimera and mixed with PBS with 5 mM EDTA, to prevent coagulation. Peripheral blood mononuclear cells were purified by density gradient centrifugation using Lympholyte-M cell separation medium (CedarLane), following the manufacturer’s instructions. The isolated mononuclear cells were transferred to cold BM buffer, pelleted and resuspended in cold BM buffer before staining for flow cytometry as indicated below.

Following verification of adequate chimerism, chimeras were euthanized, and the lymphoid organs of interest (inguinal and mesenteric lymph nodes [LNs] along with the spleen) were harvested. From the harvested lymphoid organs, lymphocytes were extracted through pestle homogenization and cells were strained through a 70 μm filter. Spleen samples were additionally resuspended in red blood cell lysis buffer (155 mM NH_4_Cl, 12 mM NaHCO_3_, 0.1 mM EDTA) for 5 min at room temperature (RT), before being diluted with cold BM buffer. All samples were centrifuged (200 g, 4°C, 5 min) and then resuspended in cold BM buffer, ready for further analysis by flow cytometry.

To a 96-well round-bottom plate were added 100 μl of the single cell suspensions, originating from either blood samples (chimerism determination and euthanasia) or harvested tissue sample (euthanasia). Each aliquoted cell suspension was mixed with 20 μl anti-CD16/CD32 antibody and incubated 5-10 min, 4°C, to block unspecific Fc-receptor binding. Each well was then added 100 μl of buffer (unstained), single antibody (for compensation controls) or antibody mixture (for flow panels) and were incubated 20 min on ice. Plates were subsequently centrifuged in swinging buckets (200 g, 4°C, 5 min) and the buffer was flicked out. Two-hundred μl BM buffer was added to each well, centrifuged, washed and flicked out again, before cells were resuspended in 200 μl BM buffer. Samples were analyzed on a Novocyte (Agilent) or an LSRFortessa (BD biosciences) flow cytometer. Only samples containing at least 100 events in the terminal GCB gate were included. This led to exclusion of a single mouse in the WT group of the TLR7 cohort, which did not display an autoimmune phenotype and consequently did not harbor splenic GC.

### Anti-dsDNA Analyses

Time-resolved immunofluorometric assay (TRIFMA) was used for measurement of total anti-dsDNA Ig. Salmon sperm dsDNA (AM9680, Invitrogen) was diluted to 500 µg/ml in PBS (Lonza), filtered through a 0.45 µm filter and further diluted to 100 µg/ml in PBS. Microtitter wells (FluoroNunc Maxisorb, Thermo Scientific) were added 100 µl of the salmon sperm DNA preparation and incubated overnight at 4°C, in a humidified box. The next day the wells were emptied, filled with 200 µl Tris-buffered saline (TBS: 10 mM Tris-HCl, 140 mM NaCl, 15 mM sodium azide (NaN_3_), pH 7.4) containing 1% w/v bovine serum albumin (BSA, A4503, Sigma-Aldrich), and incubated at RT for 1 hour. The wells were then washed three times with TBS containing 0.05% Tween-20 (TBS/Tw). All samples, controls and standards were diluted in TBS/Tw containing 5 mM EDTA and 0.1% w/v BSA. A serum pool used as standard for total anti-dsDNA IgG was diluted 1/300 and further three-fold serially diluted 7 times. Three internal controls representing high, medium and low levels of dsDNA antibodies were each diluted 1/100. The samples were diluted 1/100, 1/500 and 1/2500. All samples, controls and standards were added to plates in duplicates, 100 µl/well, then incubated for 1 hour at 37°C. Wells were emptied and washed three times with TBS/Tw before being added biotinylated Goat-anti-mouse Ig (1010-08, Southern Biotech) at 0.5 mg/ml TBS/Tw. Following incubation for 1 hour at 37°C, wells were again washed thrice with TBS/Tw, and then added 100 µl Eu^3+^-labelled streptavidin (1244-360, PerkinElmer) in TBS/Tw containing 25 μM EDTA. Following 1 hour of incubation at RT, wells were emptied, washed thrice with TBS/Tw, then developed by the addition of enhancement buffer (0.57% v/v acetic acid, 0.1% v/v Triton X-100, 1% w/v polyethylene glycol 6000, 15 μM 2-naphtoyltrifluoroacetone (2-NTA), 50 μM Tri(*n*-octyl)phosphine oxide (TOPO), pH 3.2 with potassium hydrogen phthalate) followed by 5 min vigorous agitation and finally fluorescence read-out on a Victor X5 (PerkinElmer).

### Confocal Imaging of Spleen Sections

Spleen sections were cut at 16 μm thickness on a Thermo Fisher Cryostar NX70 and mounted on Superfrost+ slides (Fisher Scientific). Sections were either fixed by addition of 1 ml acetone (Merck) and, following evaporation, subsequently rehydrated in PBS for 10 min; or by addition of 1 ml 4% paraformaldehyde (PFA) in PBS for 30 min, followed by 30 min of incubation with TBS, and finally permeabilization by a 30 min incubation with staining buffer (PBS, 2% FBS, 0.1% NaN_3_) containing 0.1% Triton-X100. The slides were then incubated overnight at 4°C with primary antibody (Ab) mixture in staining buffer. The antibody panel used for acetone fixed tissue was: CD45.1-E450, CD45.2-PE, IgD-AlexaFluor488, CD169-AlexaFluor647 (IFNAR and STING cohort 1), and for PFA fixed tissue the panel was: CD45.1-FITC, CD45.2-APC, CD169-PE, IgD-pacific blue (STING cohort 2, TLR7 and cGAS cohorts). After incubation, the slides were washed once with staining buffer for 5 min, followed by three washing steps with PBS containing 0.01% Tween-20. The slides were spot-dried and mounted using Dako Fluorescent Mounting Medium (Agilent) and 24x50mm coverslips (Hounisen). Imaging was performed on a Zeiss LSM800 Confocal Microscope with Airyscan, followed by post-processing in ImageJ.

### Molecular Modelling

The Gt point mutation I199N was evaluated for its potential impact on the unfolding Gibbs free energy change, using the multi agent stability prediction web tool MAESTRO (https://pbwww.services.came.sbg.ac.at/maestro/web/maestro/workflow). The change in the change in Gibbs free energy upon introduction of the point mutation was calculated at biochemical standard conditions (T = 298.15 K, pH = 7, P = 1 bar). The MAESTRO web tool was utilized separately on each of the monomer-strands of the murine STING-dimer structure [pdb: 4KC0 (42)], and the ratio of the unfolding equilibrium constants of the mutant, STING-Gt (I199N), K_mut_, and WT, K_WT_, was calculated for each chain of the dimeric structure in 4KC0 and averaged:


ΔΔGchainA=3.44kcalmol' ΔΔGchainB=3.44kcalmoleΔΔGchainAR·T=334, eΔΔGchainBR·T=433eΔΔGchainAR·T+ eΔΔGchainBR·T2=383

### Site-Directed Mutagenesis, STING Expression, and STING Activity Analysis

To generate a plasmid encoding STING with the Gt mutation, we performed site-directed mutagenesis (QuickChange, Qiagen) on the vector pcDNA3.1 harboring murine STING with an N-terminal Flag-Tag, following standard procedures. Primers for site-directed mutagenesis were STING-Gt-F (5’-ccaatggaaagaggttgtacagtcttcggctcc-3’) and STING-Gt-R (5’-ggagccgaagactgtacaacctctttccattgg-3’) (Eurofins MWG). After DpnI digestion of the template, the product was transformed into TOP10 *E. coli* which were plated on LB agar containing 100 µg ampicillin/ml. Several colonies were picked, amplified in LB medium containing ampicillin, and minipreps prepared. The STING-Gt mutation was verified by sequencing using the BGH-R primer (Eurofins MWG).

For expression analysis, 2.5 · 10^5^ HEK293TN cells were seeded in wells of a 12-well plate the day before transfection. Cells were transfected by adding in a pre-incubated mixture of 8 µl Lipofectamine2000 (LifeTechnologies) in Opti-MEM (Life-Technologies) and 2 µg DNA in 250 µl Opti-MEM. Twenty-four or 48 hours post-transfection, cells were washed in PBS, then lysed in 1/4 volume 4x sample buffer (10% glycerol v/v, 3% w/v SDS, 8 M urea, 60 mM Tris-HCl, pH 6.7, 0.001% w/v bromophenol blue), and added 1/10 volume 0.6 M dithiothreitol (DTT), then boiled for 3 min. Samples from transfections with STING-WT, STING-Gt, or a GFP plasmid control, along with a Precision Plus Protein Standards All Blue pre-stained marker (Bio-Rad), were then loaded onto a pre-cast 4-12% gradient Bis-Tris XT Criterion gel, which was run in XT-MOPS running buffer (Bio-Rad). The proteins were blotted onto nitrocellulose in transfer buffer (25 mM Tris, 0.192 M glycine, 20% v/v ethanol, 0.1% w/v SDS, pH 8.3) using a semi-dry blotter. The membrane was blocked in TBS/Tw 0.1% for 30 min, then incubated overnight with either rabbit anti-STING mAb (CellSignal, clone D1V5L) diluted 1/1000 in primary buffer (TBS/Tw containing 1 mg HSA/ml, 100 µg nhIgG/ml, and 1 mM EDTA), or mouse anti-FLAG mAb (SigmaAldrich, clone M2) diluted 1/5000 in primary buffer. The membrane was washed, incubated with secondary Ab in secondary buffer (TBS/Tw, 1 mM EDTA, and 100 µg human IgG/ml), and washed again before being developed with Super-Signal West Dura Extended Duration Substrate (Pierce). Images were taken using a charge-coupled device camera (ImageQuant LAS-4000; GE Healthcare) and analyzed with the Image Analysis Software supplied with the camera.

For *in vitro* STING activity analyses, HEK293T cells were seeded in 12-well plates (1 ml/well) after dilution to a density of 1·10^6^ cells/ml and incubated at 37°C and 5% CO_2_ for 24 hours. A transfection mix was made of Renilla reporter plasmid (30 ng/well), Firefly reporter plasmid (970 ng/well), cGAS plasmid (20 ng/well) and pcDNA3/STING (either WT or Gt at 100 ng/well, or none for negative control). Empty pcDNA3.1 plasmid was added to a final total DNA amount of 2000 ng/well and plain DMEM was added to a total volume of 100 μl/well. Transfection with polyethylimine (PEI, Polyscience) was performed using standard procedures. The cells were lysed with cell culture lysis reagent (200 μl/well, Promega) and the lysates were cleared by centrifugation (1 min. at 10,000 *g*). To 10 µl of cell lysate was added 32.5 μl luciferase assay substrate (Promega) and subsequently 32.5 μl Stop and Glo substrate (Promega), and luminescence was measured in relative light units (RLU) using a Wallac 1420 instrument.

### *Ex Vivo* Analyses of cGAS, STING and IFNAR Activity

Bone marrow-derived macrophages from WT, cGAS-KO and STING-Gt mice were either mock stimulated or stimulated with 2 µg dsDNA/ml for 6 hours, and the relative expression of IFN-β and β-actin measured by RT-qPCR. Bone marrow-derived macrophages from WT and IFNAR-KO mice were either mock stimulated or stimulated with 25 U IFN-β/ml for 6 hours, and the relative expression of Cxcl10 and β-actin measured by RT-qPCR.

### Data Analysis and Presentation

Flow cytometry data analysis was performed using FlowJo v. 10.7. When the congenic marker strategy allowed, the relative ratios of GCB *versus* total B cell and PC versus total B cell deriving from the compartment of interest (COI) were calculated following normalization to the total knock-out plus WT competitor compartment, according to the following formulae:


$$Relative\;GC\;participation\;in\;\%=\frac{\left(\frac{GCB[COI]}{GCB[COI]+GCB[WT]}\right)}{\left(\frac{totalB[COI]}{totalB[COI]+totalB[WT]}\right)}\cdot 100\%$$

$$Relative\;PC\;participation\;in\;\%=\frac{\left(\frac{PC[COI]}{PC[COI]+PC[WT]}\right)}{\left(\frac{totalB[COI]}{totalB[COI]+totalB[WT]}\right)}\cdot 100\%$$

Hereby, the small residual of 564Igi-derived cells, present in the total B cell but not the GCB compartment, can be ignored.

Statistical analyses were performed in GraphPad Prism v. 9 using two-way ANOVA followed by Sidak’s post-test, with significance level set at α = 0.05, as indicated in the figure legends.

Figures were rendered in Adobe Illustrator. Some figure elements were created with BioRender.com.

## Results

We set out to investigate the role of the cGAS-STING signal transduction pathway in autoreactive GCB cells using a murine mixed BM chimera model that mimics the archetypal autoimmune disease SLE (37). In this model, the presence of a single autoreactive B cell clone (clone 564Igi) is sufficient to initiate an autoreactive process that subsequently recruits the wild type (WT) B cell population. The B cells derived from the 564Igi compartment eventually are outcompeted and constitute only a minor fraction of the total B cell repertoire. Uniquely to this model, the spontaneous autoreactive GCs established by the 564Igi compartment become populated and chronically self-sustained by the non-564Igi B cells and gain independence from the initial 564Igi trigger. Six to eight weeks following reconstitution, GCs are almost exclusively (~95%) composed of WT-derived cells (37, 43). Reconstitution with a third of each of 564Igi BM, BM from a WT donor, and BM from a WT donor harboring a specified genetic defect, hence results in chimeras with two equal-sized compartments of B cells sufficient or deficient in the gene of interest. Their competitive recruitment and participation in the autoreactive GC reaction can subsequently be evaluated to elucidate the functional relevance of their intrinsic molecular differences.

### Autoreactive GC B Cells Do Not Depend on Intrinsic IFNAR Signaling

**Figure 1:**
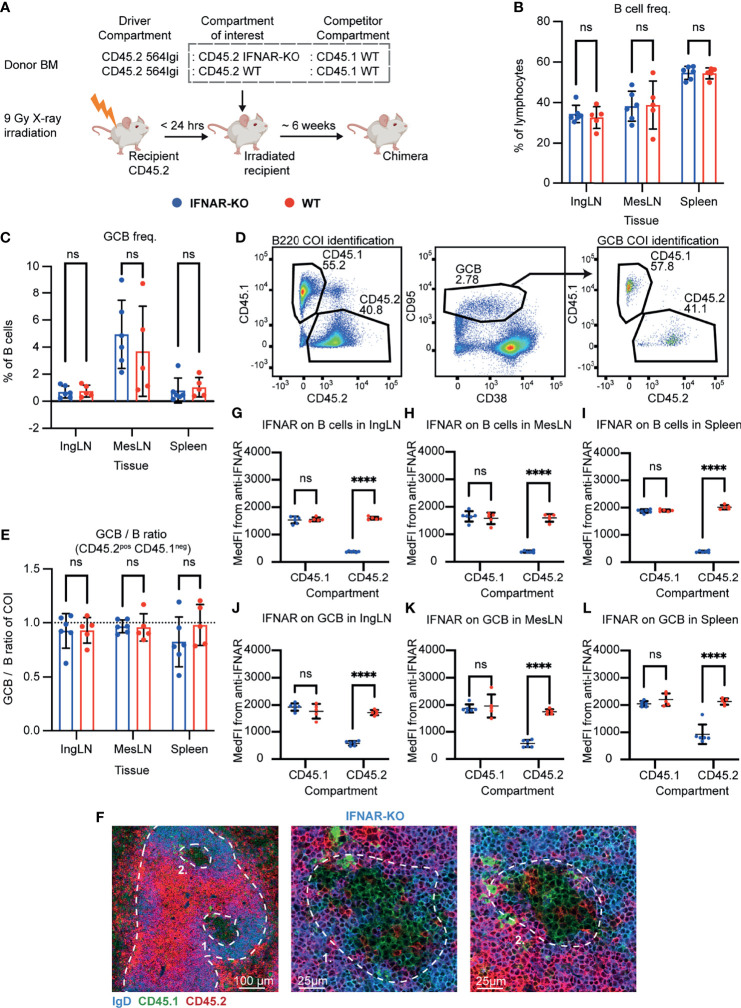
B cell intrinsic IFNAR signaling is not required for autoreactive germinal center participation. **(A)** Schematic overview of experimental setup with timeline. **(B)** B cell frequencies out of live, singlet lymphocytes, in lymphoid tissues of IFNAR-KO (blue, n = 6) and WT (red, n = 5) chimeras. Each dot represents an individual mouse and bars indicate mean +/- SD, with statistical significance given for two-way ANOVA followed by Sidak’s post-test (α = 0.05), ns = not significant. **(C)** As in **(B)**, but for GCB cell frequencies instead. **(D)** Representative example from an IFNAR-KO chimera showing gates used to define CD45.2 *vs.* CD45.1 within the B cell (left) and GCB cell (right) compartments, and the parent GCB cell gate (center). **(E)** As **(B)**, but showing relative GCB to B cell ratios for the compartment of interest (COI, CD45.2^+^CD45.1^-^). **(F)** Confocal micrographs of spleen sections from a representative IFNAR-KO chimera stained for IgD (naïve B cells, blue), CD45.1 (green), CD45.2 (red) and the marginal zone (CD169, not shown). Left, lower magnification image, with indication of the marginal zone (broken white line, based on CD169 staining, **Figure S1J**) and two GCs (numbered). Center and right panels, higher magnification images of the two GCs identified in the left panel. Channel assignments and brightness were adjusted to enhance visual clarity of micrographs. **(G)** Median fluorescence intensity of IFNAR on CD45.1 and CD45.2 B cells in inguinal lymph nodes of IFNAR-KO (blue, n = 6) and WT (red, n = 5) chimeras. Each dot represents an individual mouse and bars indicate mean +/- SD, with statistical significance given for two-way ANOVA followed by Sidak’s post-test (α = 0.05). ns = not significant, **** = p<0.0001. **(H)** as for **(G)**, but for B cells in mesenteric lymph nodes. **(I)** as for **(G)**, but for B cells in the spleen. **(J)** as for **(G)**, but for GCB cells in inguinal lymph nodes. **(K)** as for **(G)**, but for GCB cells in mesenteric lymph nodes. **(L)** as for **(G)**, but for GCB cells in the spleen.

One of the main roles of STING signaling is the induction of type I IFNs which exert their downstream effects through the IFNAR receptor. To begin to understand the potential role of STING-driven signals, we set up mixed autoreactive chimeras in which IFNAR deficient and IFNAR sufficient B cell compartments competed with each other for GC participation. To this end, lethally irradiated CD45.2 WT recipients were reconstituted with 1 part CD45.2 564Igi, 1 part CD45.2 IFNAR KO, and 1 part CD45.1 WT BM donor cells (**Figure 1A**). As a control, we set up in parallel mixed chimeras in which the CD45.2 IFNAR KO donor cells were instead replaced with CD45.2 WT BM donor cells (**Figure 1A**).

Six weeks after reconstitution, the mice were bled and the degree of chimerism analyzed by flow cytometry. Both groups displayed normal and comparable levels of B cells, CD4 and CD8 T cells (**Figures S1A–C**). In the T cell compartment, approximately two thirds of the cells carried CD45.2, and one third CD45.1, in agreement with a near-complete ablation of the recipient compartment, and an equal representation of each of the three donor compartments (**Figures S1D, E**). In the B cell compartment, approximately half of the B cells were CD45.2 and half CD45.1 (**Figure S1F**), fitting with the previously noted negative selection of 564Igi cells upon reconstitution (37). In further agreement with this notion, only a low residual frequency of 9D11 positive cells carrying the knock-in receptor of the 564Igi compartment remained (**Figure S1G**). Despite this, circulating anti-dsDNA autoantibodies were detectable (**Figure S1H**). Taken together, this demonstrated that loss of IFNAR did not impair reconstitution of the major lymphocyte subsets, and that the absence of IFNAR signaling competence in one third of the hematopoietic lineage did not prevent the autoreactive phenotype of the model. To verify the functional effect of the IFNAR-KO, we generated *ex vivo* bone marrow-derived macrophages from IFNAR-KO and WT mice, stimulated these with IFN-β, and measured the expression of Cxcl10 relative to β-actin. WT derived macrophages produced Cxcl10 in response to IFN-β, whereas IFNAR-KO derived macrophages did not (**Figure S1I**).

To investigate the B cell intrinsic role of IFNAR in GC participation, the chimeras were euthanized and spleens, inguinal and mesenteric LNs harvested for flow analysis. Normal and comparable levels of B cells were found in the IFNAR-KO and internal control (WT) setup (**Figure 1B**). Both groups displayed appreciable (~1%) GCB cell frequencies in the spleen and IngLN, commensurate with the autoimmune phenotype, and robust GCB cell frequencies in the MesLN, likely as an additive effect of the autoimmune phenotype and the response to the gut microbiota at this anatomical location (**Figure 1C**). To directly determine the competitive potential of B cells deficient in IFNAR for GC participation, we gated on the CD45.2 cells in the GC compartment (CD38^lo^, CD95^hi^ of B cells) and related this to the frequency of CD45.2 cells in the total B cell pool (B220^+^ of live, singlet lymphocytes) (**Figure 1D**). If the B cell intrinsic loss of IFNAR significantly impaired GC participation, we would expect to see an underrepresentation of cells from the compartment of interest (COI) among the GCB cells, relative to the WT CD45.1 competitor cells, but only when the COI was IFNAR KO and not in the WT CD45.2 control chimeras. As can be seen in **Figure 1E**, the relative ratios of the COI GCB to COI B cells were comparable between the two groups across tissues. We confirmed this observation by microscopy, demonstrating the presence of both CD45.1 and CD45.2 cells in the GC (**Figures 1F**, **S1J**). Therefore, the presence or absence of IFNAR did not appear to impact the ability of GCB cells to participate in the autoreactive GC responses of spleen and IngLN, nor in the mixed responses of the MesLN. Yet the possibility remained that the CD45.2 cells observed were not derived from the COI, but from 564Igi or WT recipient cells, which were also CD45.2.

As we could not uniquely assign the CD45.2 cells to the IFNAR KO compartment, because they were indistinguishable from 564Igi and recipient compartments, we sought to experimentally verify that the cells of interest were indeed IFNAR KO. To this end, we stained the cells with an anti-IFNAR antibody and determined the Median Fluorescence Intensity (MedFI) in the total B cell and GCB cell compartments. As can be seen, CD45.1 WT cells from both groups displayed robust expression of IFNAR in both compartments (**Figures 1G–L**). However, in the IFNAR KO group, CD45.2 cells displayed a significantly reduced expression of IFNAR, whereas the expression level of CD45.2 cells in the control WT group was on par with that of the CD45.1 cells. These findings were recapitulated by gating of IFNAR positive *versus* negative cells across the CD45.1 and CD45.2 populations (**Figures S1K–P**). Accordingly, we concluded that the bulk of CD45.2 cells present in the total B cell and GCB cell pool from IFNAR KO chimeras were in fact derived from the IFNAR KO bone marrow donor, and were indeed IFNAR deficient. Yet these cells were present to a similar extent in GC as the WT cells, thus directly demonstrating their independence of intrinsic IFNAR signals for GC participation.

### Autoreactive GC B Cells Do Not Depend on Intrinsic STING Signaling

Type I IFN production and IFNAR signaling is only one of the effectors of cGAS-STING signaling, which diverges into four independent downstream functionalities: IRF3 activation, NF-κB and MAPK pathway activation, autophagy and lysosomal cell death (LCD). To more directly interrogate the potential role of STING-driven signals, which could also be independent of IFNAR, we set up mixed autoreactive chimeras in which STING deficient and STING sufficient B cell compartments competed with each other for GC participation. To this end, we employed the STING *Golden-ticket* (STING-Gt) mutant (I199N), which is generally considered a functional STING knock-out (44).

**Figure 2:**
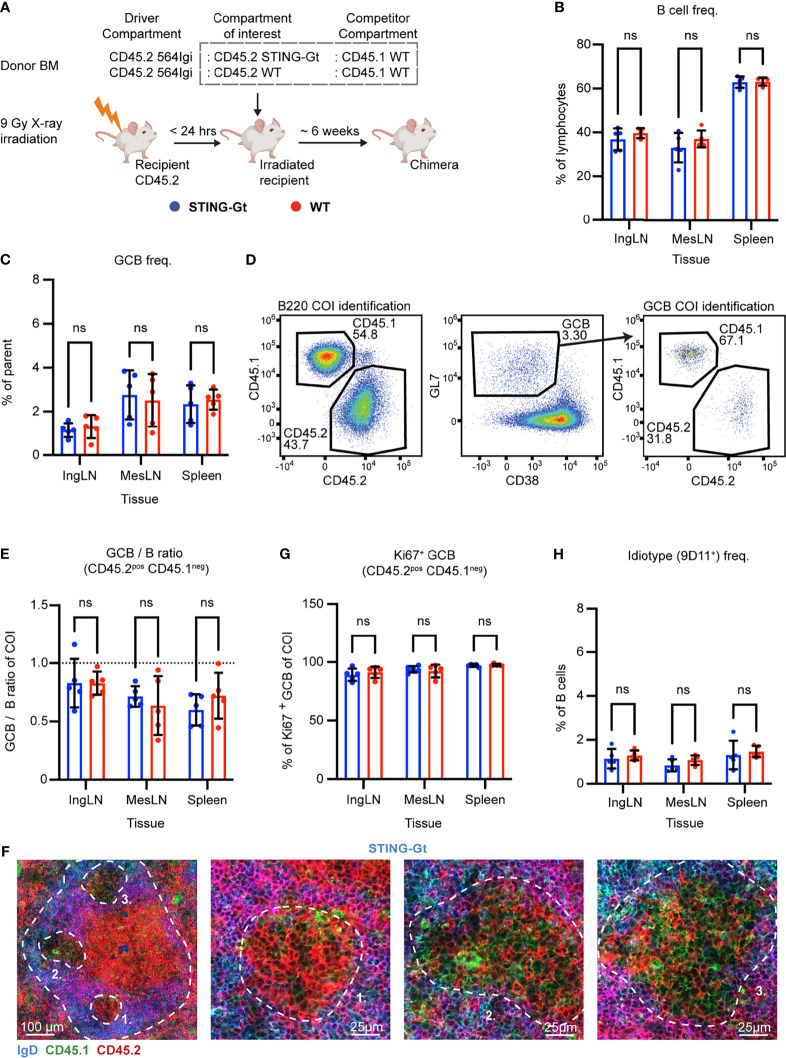
B cell intrinsic STING signaling is not required for autoreactive germinal center participation. **(A)** Schematic overview of experimental setup with timeline. **(B)** B cell frequencies out of live, singlet lymphocytes, in lymphoid tissues of STING-Gt (blue, n = 5) and WT (red, n = 5) chimeras. Each dot represents an individual mouse and bars indicate mean +/- SD, with statistical significance given for two-way ANOVA followed by Sidak’s post-test (α = 0.05), ns = not significant. **(C)** As in **(B)**, but for GCB cell frequencies instead. **(D)** Representative example from a STING-Gt chimera showing gates used to define CD45.2 *vs.* CD45.1 within the B cell (left) and GCB cell (right) compartments, and the parent GCB cell gate (center). **(E)** As **(B)** but showing relative GCB to B cell ratios for the compartment of interest (COI, CD45.2^+^CD45.1^-^). **(F)** Confocal micrographs of spleen sections from a representative STING-Gt chimera stained for IgD (naïve B cells, blue), CD45.1 (green), CD45.2 (red) and the marginal zone (CD169, not shown). Left, lower magnification image, with indication of the marginal zone (broken white line, based on CD169 staining, **Figure S2G**) and three GCs (numbered). Subsequent panels present higher magnification images of each of the three numbered GCs in the left panel. Channel assignments and brightness were adjusted to enhance visual clarity of micrographs. **(G)** As **(B)**, but for Ki67 positive frequency within the GCB cell compartment of interest (COI, CD45.2^+^CD45.1^-^). **(H)** As **(B)**, but for 564Igi idiotype (9D11^+^) frequencies of B cells.

Similar to the IFNAR KO chimeras, lethally irradiated CD45.2 WT recipients were reconstituted with 1 part CD45.2 564Igi, 1 part CD45.2 STING-Gt, and 1 part CD45.1 WT BM donor cells (**Figure 2A**). Again, as a control, we in parallel set up mixed chimeras, in which the CD45.2 STING-Gt donor cells were instead replaced with CD45.2 WT BM donor cells (**Figure 2A**).

Blood samples were taken from the chimeras six weeks after reconstitution, and analyzed for the degree of chimerism by flow cytometry. Both groups displayed normal and comparable levels of B cells and T cells (**Figures S2A, B**). In the T cell compartment, approximately two thirds of the cells carried CD45.2, and one third CD45.1, again in agreement with a near-complete ablation of the recipient compartment, and an equal representation of each of the three donor compartments (**Figure S2C**). In the B cell compartment, in agreement with the previously noted negative selection of 564Igi cells upon reconstitution, approximately half of the B cells were CD45.2 and half CD45.1, albeit with a slight underrepresentation of the STING compartment (**Figure S2D**). Only a low residual frequency of 9D11 positive cells carrying the knock-in receptor of the 564Igi compartment remained (**Figure S2E**). Despite this, a robust level of circulating anti-dsDNA autoantibodies was detectable (**Figure S2F**). As for IFNAR, this demonstrated that loss of STING did not impair reconstitution of the major lymphocyte subsets, and that the absence of STING signaling competence in one third of the hematopoietic lineage did not limit the autoreactive phenotype of the model.

To investigate the B cell intrinsic role of STING in GC participation, the mice were euthanized and spleens, inguinal and mesenteric LNs harvested for flow analysis. Normal and comparable levels of B cells were found in the STING-Gt and internal control (WT) setup (**Figure 2B**). Both groups displayed appreciable (~1-3%) GCB cell frequencies in the spleen and IngLN, commensurate with the autoimmune phenotype, and in the MesLN, likely in response to the gut microbiota (**Figure 2C**). To directly determine the competitive potential of B cells deficient in STING for GC participation, we gated on the CD45.2 cells in the GC compartment (CD38^lo^, GL7^hi^ of B cells) and related this to the frequency of CD45.2 cells in the total B cell pool (B220^+^ of live, singlet lymphocytes) (**Figure 2D**). If the B cell intrinsic loss of STING significantly impaired GC participation, we would expect to see an underrepresentation of cells from the COI among the GCB cells, relative to the WT CD45.1 cells, but only when the COI was STING KO and not in the WT CD45.2 control chimeras. As can be seen in **Figure 2E**, the relative ratios of the COI GCB to COI B cells were comparable between the two groups across tissues. We confirmed this observation by microscopy, demonstrating the presence of both CD45.1 and CD45.2 cells in the GC (**Figures 2F**, **S2G**). Therefore, the presence or absence of STING did not appear to impact the ability of GCB cells to participate in the autoreactive GC responses of spleen and IngLN, nor in the mixed responses of the MesLN.

STING has additionally been suggested to regulate cell death signaling in lymphocytes (45), and since a hallmark of GCB responses is rapid proliferation regulated by high rates of controlled apoptosis, we investigated if the degree of proliferating GCB cells varied between the compartments with either a sufficient or deficient STING function. To this end, we performed intracellular staining for the nuclear marker of proliferation, Ki67. We did not observe any variation in the proportion of proliferating GCB that correlated to STING-functionality (**Figure 2G**).

Based on our experience with the IFNAR chimeras, we surmised that recipient cells were efficiently ablated and that 564Igi donor cells did not contribute significantly to the reconstituted compartments at equilibrium. Although it appeared unlikely that the STING BM chimera setup would behave fundamentally differently from that of the IFNAR chimeras, the possibility did remain that the CD45.2 cells observed were derived from the indistinguishable 564Igi and recipient compartments. However, unlike for IFNAR, we did not have antibodies available to stain directly for STING in the flow cytometric analyses. Furthermore, it was unclear whether STING-Gt B cells would express non-functional STING, and hence could be stained by such an antibody, despite being functionally deficient. Therefore, as a first step towards excluding participation of the 564Igi derived cells, we stained with the 9D11 anti-idiotypic antibody and observed only a low frequency of these cells across the tissues of interest (**Figure 2H**). However, this still fell short of excluding a contribution of hypermutated 564Igi-derived cells that could have lost 9D11 positivity, as well as a potential residual from the recipients.

**Figure 3:**
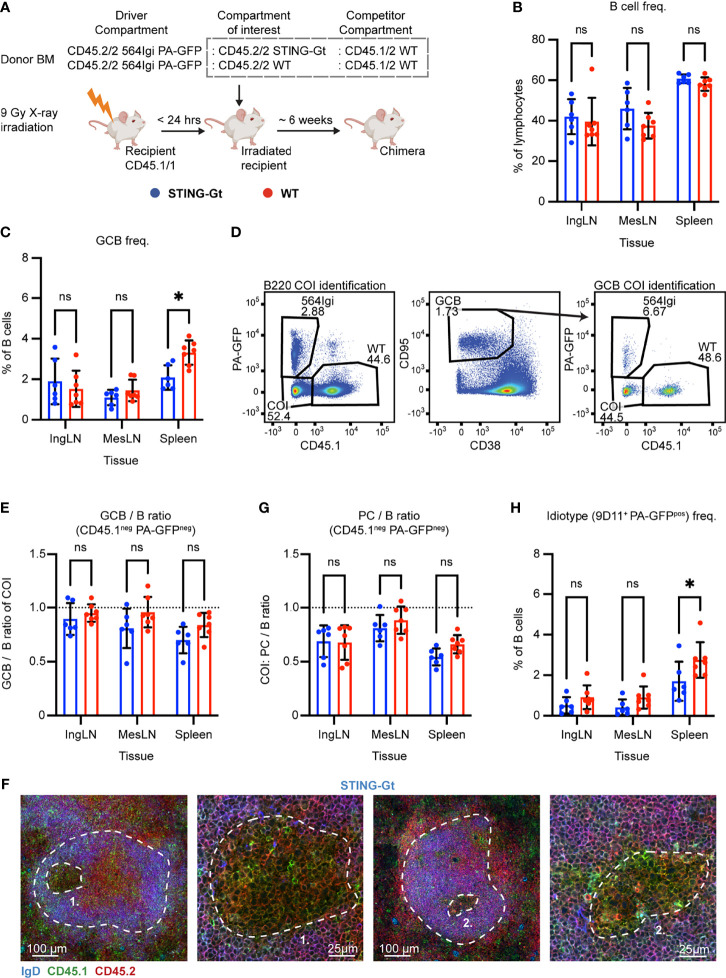
Unique identification of STING-Gt cells recapitulates the finding that B cell intrinsic STING signaling is not required for autoreactive germinal center participation. **(A)** Schematic overview of experimental setup with timeline. **(B)** B cell frequencies out of live, singlet lymphocytes, in lymphoid tissues of STING-Gt (blue, n = 6) and WT (red, n = 7) chimeras. Each dot represents an individual mouse and bars indicate mean +/- SD, with statistical significance given for two-way ANOVA followed by Sidak’s post-test (α = 0.05), ns = not significant, * = p<0.05. **(C)** As in **(B)**, but for GCB cell frequencies instead. **(D)** Representative example from a STING-Gt chimera showing gates used to define CD45.1 *vs.* PA-GFP within the B cell (left) and GCB cell (right) compartments, and the parent GCB cell gate (center). **(E)** As **(B)** but showing relative GCB to B cell ratios for the compartment of interest (COI, CD45.1^-^ PA-GFP^-^). **(F)** Confocal micrographs of spleen sections from a representative STING-Gt chimera stained for IgD (naïve B cells, blue), CD45.1 (green), CD45.2 (red) and the marginal zone (CD169, not shown). From left, lower magnification image with indication of the marginal zone (broken white line, based on CD169 staining, **Figure S3J**) and a GC (numbered), followed by a higher magnification image of that GC. Then another lower magnification image with indication of the marginal zone (broken white line, based on CD169 staining, S3J), and a GC (numbered), followed by a higher magnification image of that GC. Channel assignments and brightness were adjusted to enhance visual clarity of micrographs. **(G)** As **(E)** but showing relative PC (plasma blast/cell, CD138^hi^ within live, singlet lymphocytes) to B cell ratios for the compartment of interest (COI, CD45.1^-^ PA-GFP^-^). **(H)** As **(B)**, but for 564Igi idiotype (9D11^+^) frequencies of PA-GFP positive B cells.

To ensure that the lack of any observed effect of STING deficiency was not an artefact due to residual 564Igi or WT recipient cells, we repeated the experiment in a manner where we could definitively discriminate the three donor compartments and the recipient compartment. Accordingly, lethally irradiated CD45.1/1 WT recipients were reconstituted with 1 part CD45.2/2 564Igi cells additionally carrying photoactivatable green fluorescent protein (PA-GFP) as a congenic marker, 1 part CD45.2/2 STING-Gt, and 1 part CD45.1/2 WT BM donor cells (**Figure 3A**). Control (WT) chimeras were again set up in parallel, in which the CD45.2/2 STING-Gt donor cells were instead replaced with CD45.2/2 WT BM donor cells (**Figure 3A**).

We took blood samples six weeks after reconstitution and analyzed the degree of chimerism by flow cytometry. Normal and comparable levels of B cells, CD4 and CD8 T cells were observed in both groups (**Figures S3A–C**). In the T cell compartment, approximately two thirds of the cells were CD45.1 negative, half of these PA-GFP^pos^ the other half PA-GFP^neg^, while one third carried CD45.1. This again agreed with a near-complete ablation of the recipient compartment, and an equal representation of each of the three donor compartments (**Figures S3D–F**). In the B cell compartment, approximately half of the B cells were CD45.1 PA-GFP double negative and half CD45.1 positive, whereas barely any CD45.1^neg^ PA-GFP^pos^ 564Igi-derived cells were detected (**Figure S3G**), in agreement with the previously noted negative selection of 564Igi cells upon reconstitution. In further agreement with this notion, only a low residual frequency of 9D11 positive cells carrying the knock-in receptor of the 564Igi compartment remained (**Figure S3H**). A robust level of circulating anti-dsDNA autoantibodies was detectable in both groups (**Figure S3I**).

The mice were euthanized and spleens, inguinal and mesenteric LNs harvested for flow analysis. Normal and comparable levels of B cells were found in the STING-Gt and internal control (WT) setup (**Figure 3B**). Although both groups displayed appreciable (~1-3%) GCB cell frequencies in the spleen and IngLN, commensurate with the autoimmune phenotype, we noted that the GC frequency was slightly higher in the spleen of the WT control group. Notwithstanding this difference, both groups did have a robust GCB phenotype (**Figure 3C**). Accordingly, we again directly interrogated the competitive potential of B cells deficient in STING for GC participation, by gating on the CD45.1 PA-GFP double negative cells in the GC compartment (CD38^lo^, CD95^hi^ of B cells) and related this to the frequency of CD45.1 PA-GFP double negative cells in the total B cell pool (B220^+^ of live, singlet lymphocytes) (**Figure 3D**). As can be seen in **Figure 3E**, the relative ratios of the COI GCB to COI B cells were comparable between the two groups across tissues. We confirmed this observation by microscopy, demonstrating the presence of both CD45.1/2 and CD45.2/2 cells in the GC (**Figures 3F**, **S3J**). The lack of difference was similarly recapitulated in the PC compartment (**Figure 3G**). The idiotype frequency in the spleen was slightly higher for the WT group, as compared to the STING-Gt group (**Figure 3H**), potentially explaining the slight difference in GC magnitude in this tissue between groups (**Figure 3C**). Importantly, however, the congenic marker strategy employed in this experiment allowed exclusion of any 564Igi contribution in our flow cytometry results. In line with the findings from our previous STING cohort, this confirmed that the presence or absence of STING did not impact the ability of GCB cells to participate in the autoreactive GC responses of spleen and IngLN, nor in the mixed responses of the MesLN.

Taken together, our results indicated that STING deficiency did not impair autoreactive GCB cell fitness. This was at odds with a recent report demonstrating a function of STING in regulating the BCR, and also impacting GCB cells (33). That study was based on a complete STING knock-out, and hence we considered the possibility that STING-Gt could retain some level of functionality, although it has been used extensively in the literature as a functional knock-out. We evaluated the potential impact of the Gt point mutation I199N on the unfolding Gibbs free energy change, using MAESTRO (46). This prediction revealed that the mutant is approximately 383 times more likely to be unfolded at biochemical standard conditions than the WT variant of STING, indicating a destabilizing contribution of the STING-Gt mutation I199N on the process of STING monomer-folding. Notwithstanding these theoretical considerations, we decided to also experimentally investigate the impact of the STING-Gt mutation on the stability of the protein. To this end, we employed a wild-type murine STING expression construct with an N-terminal FLAG tag, and generated the STING-Gt variant by site-directed mutagenesis. We subsequently transfected HEK293 cells with the two constructs, or a control GFP construct, and assessed the expression level by Western blotting. As can be seen in **Figure S3K**, using an anti-FLAG antibody for detection, we saw a reduced level of expression of STING-Gt relative to wild-type STING. This finding was recapitulated upon detection with an antibody to STING (**Figure S3L**). Nonetheless, STING-Gt was detectable, and hence not completely absent, raising the question of residual functionality. We subsequently tested the activity of STING WT and Gt in a luciferase assay relying on co-expression with cGAS in HEK293 cells. As shown in **Figure S3M**, whereas WT STING was active in this assay, STING-Gt did not have any detectable activity above background. These findings directly recapitulated the original observations of Sauer and colleagues (44). To verify that the STING-Gt mice in our colony were in fact devoid of STING activity, we furthermore generated *ex vivo* bone marrow-derived macrophages and stimulated these with dsDNA. WT derived macrophages produced IFN-β in response to dsDNA stimulation, whereas STING-Gt derived macrophages did not (**Figure S3N**). Taken together, our results verified that STING-Gt faithfully recapitulates a functional STING knock-out.

### Autoreactive GC B Cells Depend Critically on Intrinsic TLR7 Signaling

**Figure 4:**
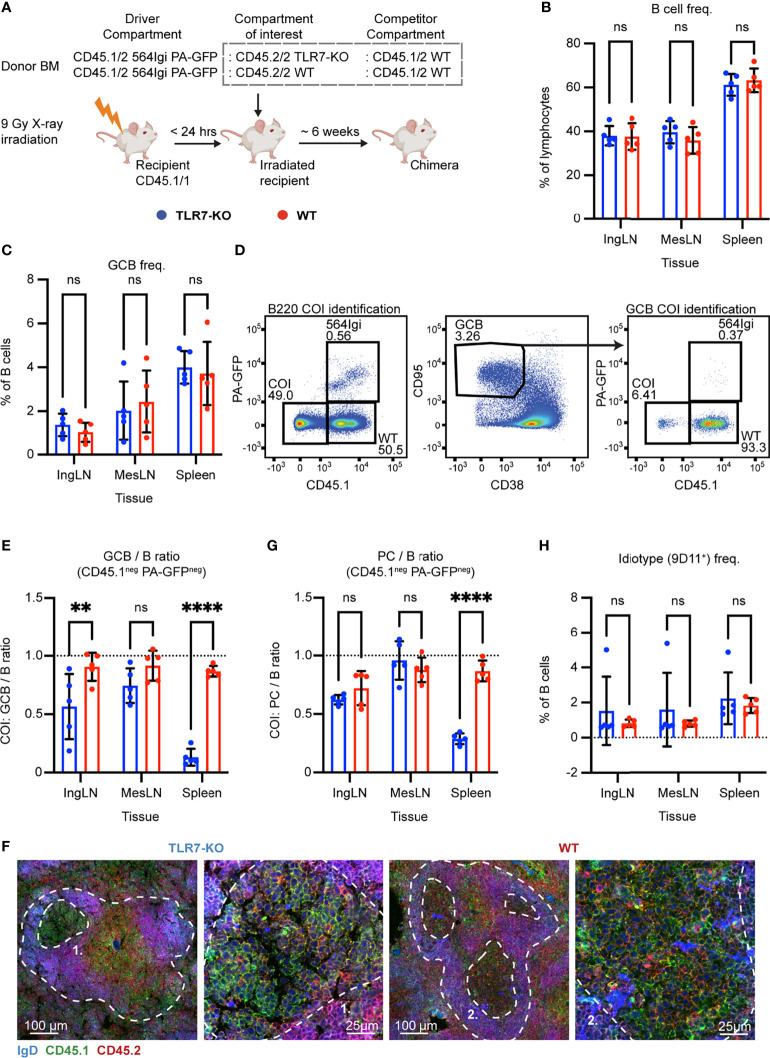
B cell intrinsic TLR7 signaling is critical for autoreactive germinal center participation. **(A)** Schematic overview of experimental setup with timeline. **(B)** B cell frequencies out of live, singlet lymphocytes, in lymphoid tissues of TLR7-KO (blue, n = 5) and WT (red, n = 5) chimeras. Each dot represents an individual mouse and bars indicate mean +/- SD, with statistical significance given for two-way ANOVA followed by Sidak’s post-test (α = 0.05), ns = not significant, ** = p<0.01, **** = p<0.0001. **(C)** As in **(B)**, but for GCB cell frequencies instead. **(D)** Representative example from a TLR7-KO chimera showing gates used to define CD45.1 *vs.* PA-GFP within the B cell (left) and GCB cell (right) compartments, and the parent GCB cell gate (center). **(E)** As **(B)** but showing relative GCB to B cell ratios for the compartment of interest (COI, CD45.1^-^ PA-GFP^-^). **(F)** Confocal micrographs of spleen sections from a representative TLR7-KO chimera and WT control chimera stained for IgD (naïve B cells, blue), CD45.1 (green), CD45.2 (red) and the marginal zone (CD169, not shown). Left two panels originate from a TLR7-KO chimera, lower magnification image with indication of the marginal zone (broken white line, based on CD169 staining, **Figure S4J**) and a GC (numbered), followed by higher magnification image of that GC. Right two panels, like those on the left but originating from a WT chimera instead. Channel assignments and brightness were adjusted to enhance visual clarity of micrographs. **(G)** As **(E)** but showing relative PC (plasma blast/cell, CD138^hi^ within live, singlet lymphocytes) to B cell ratios for the compartment of interest (COI, CD45.1^-^ PA-GFP^-^). **(H)** As **(B)**, but for 564Igi idiotype (9D11^+^) frequencies of B cells.

Having observed that neither IFNAR nor STING had an impact on GCB cell fitness in the mixed chimera model, we considered the possibility that the model did not faithfully recapitulate the autoreactive epitope spreading originally observed. Although it was previously demonstrated that the mixed chimera model is a robust model for interrogating signal pathways essential for autoreactive GCB cell function (37), the establishment of the model in another animal facility and setting could potentially preclude 1:1 extrapolation of the phenotype. Hence, to provide an internal control in our setup to validate the robustness of the model, we set up a similar mixed chimera cohort with internal competition between TLR7 sufficient and deficient cells, as TLR7 was previously demonstrated to significantly impact GCB cell fitness (37). Accordingly, myeloablated CD45.1/1 WT recipients were reconstituted with 1 part CD45.1/2 564Igi donor cells, which additionally carried PA-GFP, 1 part CD45.2/2 TLR7 KO, and 1 part CD45.1/2 WT donor BM cells (**Figure 4A**). Again, as a control, we set up in parallel mixed chimeras, in which the CD45.2/2 TLR7 KO donor cells were instead substituted with CD45.2/2 WT BM donor cells (**Figure 4A**).

Six weeks post reconstitution, we evaluated the degree of chimerism in the blood by flow cytometry. Normal and comparable levels of B cells, CD4 and CD8 T cells were observed in both groups (**Figures S4A–C**). In the T cell compartment, approximately two thirds of the cells carried CD45.1, half of these PA-GFP^pos^ the other half PA-GFP^neg^, and one third were CD45.2/2, again in agreement with a near-complete ablation of the recipient compartment, and an equal representation of each of the three donor compartments (**Figures S4D–F**). In the B cell compartment, approximately half of the B cells were CD45.2 only, half CD45.1 positive, and very few of the CD45.1 positive cells were PA-GFP^pos^ (**Figure S4G**), in agreement with the previously noted negative selection of 564Igi cells upon reconstitution. In further agreement with this notion, only a low residual frequency of 9D11 positive cells carrying the knock-in receptor of the 564Igi compartment remained (**Figure S4H**). Despite this, an appreciable level of circulating anti-DNA autoantibodies was detectable (**Figure S4I**). As for IFNAR and STING, this demonstrated that loss of TLR7 did not impair reconstitution of the major lymphocyte subsets, and that the absence of TLR7 signaling competence in one third of the hematopoietic lineage did not limit the autoreactive phenotype of the model.

The chimeras were euthanized, and we investigated the spleens, inguinal and mesenteric LNs. Normal and comparable levels of B cells were found in the TLR7-KO and internal control (WT) setup (**Figure 4B**). GC B cells were present in significant numbers, upwards of 4% in the spleen and ~2% in the inguinal and mesenteric LNs (**Figure 4C**). We again gated out the COI (identified by their CD45.2/2 alleles, being CD45.1^neg^ and PA-GFP^neg^) within the total B cell and GCB cell pools (**Figure 4D**), and observed a dramatic and statistically significant disadvantage for TLR7 deficient B cells, in their participation in GC reactions of spleen and skin-draining inguinal lymph nodes (**Figure 4E**). This effect was, however, not observed in the mesenteric LNs, in agreement with previous observations (37), likely as a consequence of the diverse pathways driving GC responses towards the microbiota in this anatomical location. Hence, our observations faithfully recapitulated the prior observations in this model (37). Using confocal microscopy, we confirmed the finding that TLR7-KO cells were largely excluded from the GC (**Figures 4F**, **S4J**). We furthermore observed a significantly lowered PC output from TLR7-deficient B cells in the spleen, compared to the TLR7-sufficient cells, when looking at the relative ratios of PCs to B cells from the COI (**Figure 4G**). This indicated a functional impact of the disability of TLR7 deficient B cells in GCs, in turn causing a lowered plasma cell output from this compartment. The frequency of idiotype positive cells was low across tissues in both groups (**Figure 4H**). Taken together, these observations verified the fidelity of the model.

### Autoreactive GC B Cells Do Not Depend on Intrinsic cGAS-Mediated Sensing

**Figure 5:**
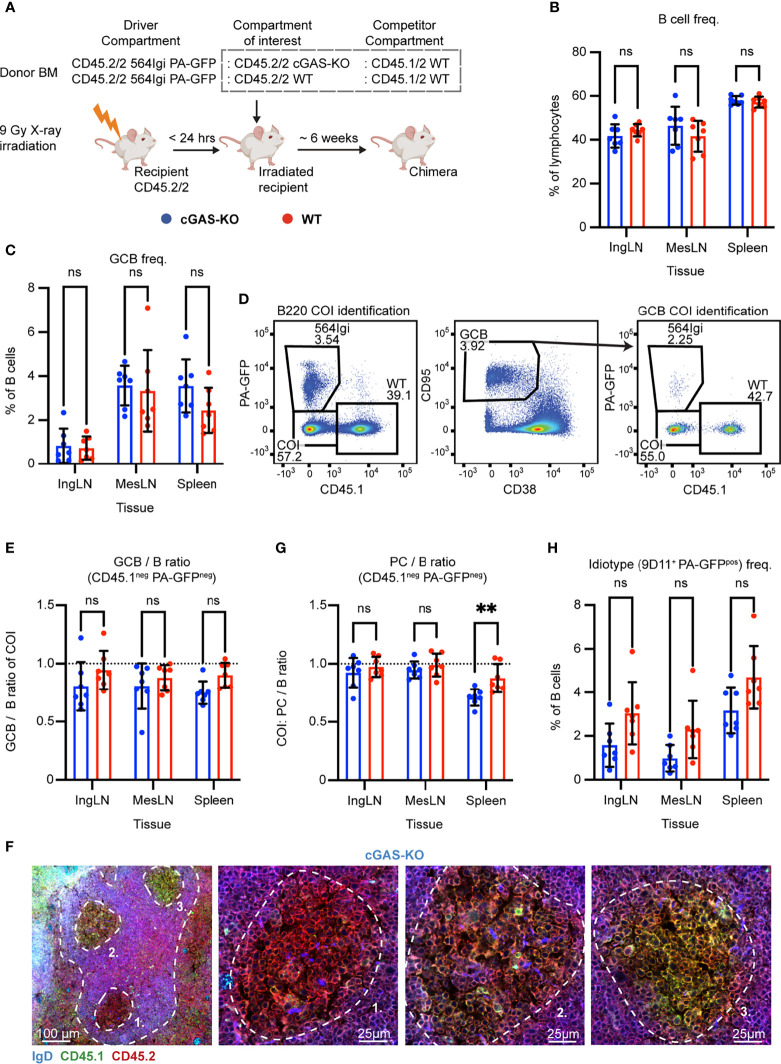
B cell intrinsic cGAS signaling is not required for autoreactive germinal center participation but marginally impacts PC output in the spleen. **(A)** Schematic overview of experimental setup with timeline. **(B)** B cell frequencies out of live, singlet lymphocytes, in lymphoid tissues of cGAS-KO (blue, n = 7) and WT (red, n = 7) chimeras. Each dot represents an individual mouse and bars indicate mean +/- SD, with statistical significance given for two-way ANOVA followed by Sidak’s post-test (α = 0.05), ns = not significant, ** = p<0.01. **(C)** As in **(B)**, but for GCB cell frequencies instead. **(D)** Representative example from a cGAS-KO chimera showing gates used to define CD45.1 *vs.* PA-GFP within the B cell (left) and GCB cell (right) compartments, and the parent GCB cell gate (center). **(E)** As **(B)** but showing relative GCB to B cell ratios for the compartment of interest (COI, CD45.1^-^ PA-GFP^-^). **(F)** Confocal micrographs of spleen sections from a representative cGAS-KO chimera stained for IgD (naïve B cells, blue), CD45.1 (green), CD45.2 (red) and the marginal zone (CD169, not shown). Left, lower magnification image, with indication of the marginal zone (broken white line, based on CD169 staining, **Figure S5K**) and three GCs (numbered). Subsequent panels present higher magnification images of each of the three numbered GCs in the left panel. Channel assignments and brightness were adjusted to enhance visual clarity of micrographs. **(G)** As **(E)** but showing relative PC (plasma blast/cell, CD138^hi^ within live, singlet lymphocytes) to B cell ratios for the compartment of interest (COI, CD45.1^-^ PA-GFP^-^). **(H)** As **(B)**, but for 564Igi idiotype (9D11^+^) frequencies of PA-GFP positive B cells.

STING works through the upstream signal mediator cGAS, a nucleotidyl transferase which initiates the cGAS-STING pathway upon dsDNA binding. To corroborate our findings and further test the role of the STING pathway in an independent genetic model, we set up mixed chimeras in which cGAS sufficient or deficient cells were competing with each other. Lethally irradiated CD45.2/2 WT recipients were reconstituted with 1 part CD45.2/2 564Igi carrying PA-GFP, 1 part CD45.2/2 cGAS KO, and 1 part CD45.1/2 WT BM donor cells (**Figure 5A**). As a control, we set up in parallel mixed chimeras, in which CD45.2/2 WT BM donor cells were substituted for the CD45.2/2 cGAS KO donor cells (**Figure 5A**).

The chimeric phenotype was verified by flow cytometry on blood samples six weeks post reconstitution. Both groups displayed normal and comparable levels of B cells, CD4 and CD8 T cells (**Figures S5A–C**). In the T cell compartment, approximately two thirds of the cells carried CD45.2 only, half of these PA-GFP^pos^, the other half PA-GFP^neg^, and one third were CD45.1/2, again in agreement with a near-complete ablation of the recipient compartment, and an equal representation of each of the three donor compartments (**Figures S5D–F**). In the B cell compartment, approximately half of the B cells were CD45.2/2 and half CD45.1/2, and very few of the CD45.2/2 cells were PA-GFP^pos^ (**Figure S5G**), in agreement with the previously noted negative selection of 564Igi cells upon reconstitution. In further agreement with this notion, only a low residual frequency of 9D11 positive cells carrying the knock-in receptor of the 564Igi compartment remained, and slightly more in the WT setup (**Figure S5H**). An appreciable level of circulating anti-dsDNA autoantibodies was detectable in the cGAS chimeras, although the level was significantly higher in the WT group (**Figure S5I**). As for IFNAR, STING and TLR7, this demonstrated that loss of cGAS did not impair reconstitution of the major lymphocyte subsets, and that the absence of cGAS signaling competence in one third of the hematopoietic lineage did not prevent the autoreactive phenotype of the model. To verify the functional effect of the cGAS-KO, we generated *ex vivo* bone marrow-derived macrophages from cGAS-KO and WT mice, stimulated these with dsDNA, and measured the expression of IFN-β relative to β-actin. WT derived macrophages produced IFN-β in response to dsDNA, whereas cGAS-KO derived macrophages did not (**Figure S5J**).

To investigate the B cell intrinsic role of cGAS in GC participation, the chimeras were euthanized and spleens, inguinal and mesenteric LNs harvested for flow analysis. Normal and comparable levels of B cells were found in the cGAS-KO and internal control (WT) setup (**Figure 5B**). Both groups displayed appreciable (~1-4%) GCB cell frequencies in the inguinal LN, mesenteric LN and spleen (**Figure 5C**). To directly determine the competitive potential of B cells deficient in cGAS for GC participation, we gated on the CD45.1 PA-GFP double negative cells in the GC compartment (CD38^lo^, CD95^hi^ of B cells) and related this to the frequency of CD45.1 PA-GFP double negative cells in the total B cell pool (B220^+^ of live, singlet lymphocytes) (**Figure 5D**). As can be seen in **Figure 5E**, the relative ratios of the COI GCB to COI B cells were comparable between the two groups across tissues. We confirmed this observation by microscopy, demonstrating the presence of both CD45.1/2 and CD45.2/2 cells in the GC (**Figures 5F**, **S5K**). We furthermore observed a marginally, but significantly, lowered PC output from cGAS-deficient B cells in the spleen, compared to the cGAS-sufficient setup, when looking at the relative ratios of PCs to B cells from the COI (**Figure 5G**). The frequency of idiotype cells across tissues did not differ significantly between the two groups (**Figure 5H**). Despite the slight effect of cGAS deficiency on PC output, the presence or absence of cGAS clearly did not impact the ability of GCB cells to participate in the autoreactive GC responses of spleen and inguinal LN, nor in the mixed responses of the mesenteric LN.

## Discussion

Here, we investigated the B cell intrinsic role of the cGAS-STING pathway in autoreactive GCB cell responses. We leveraged a unique mouse model initiated by a single B cell clone reactive with DNA and ribonuclear antigens, and displaying rampant epitope spreading in the WT compartment. In this model, it is possible to isolate the B cell intrinsic effect of genetic alterations in a direct competitive scenario within the WT compartment. Importantly, because the 564Igi BM contributes equally to the non-B cell compartments, at least two thirds of non-B cells, and all recipient-derived stromal cells, do not carry the specified genetic defect, rendering the environment sufficient. Accordingly, these chronic and WT-sustained GCs carry a unique possibility to investigate a B cell intrinsic influence of a signaling parameter in an internally controlled competitive scenario. The model furthermore includes physiological GC characteristics such as a pauciclonal evolution (47) and T cell dependence (37).

Prior studies on the B cell intrinsic roles of STING are limited, and have yielded seemingly contradictory results. Using the Mb1-Cre STING^flx/flx^ model, Walker and colleagues found that B cell intrinsic STING signaling synergizes with BCR signals to promote antibody responses (35). In direct contrast to these findings, a recent report demonstrated that B cells carrying constitutively activated STING are less capable of responding to BCR activation, and consequently, that B cell-specific STING KO mice (CD19-Cre STING^flx/flx^) produced significantly more antigen-specific plasma cells upon T-independent antigen immunization (34). In agreement with the second of these studies, Jing et al. reported that STING negatively regulates BCR signaling by inhibiting the activation of CD19 and Btk, but surprisingly, in line with the first of the studies they also found that STING is required for an efficient humoral immune response elicited by T-dependent antigens. They suggested this to be due to a B cell intrinsic effect, although it was investigated in a global STING KO model (33).

We did not observe any role for STING in autoreactive GCB cell responses, neither through direct abrogation of STING activity through the STING-Gt mutation, nor by complete knock-out of the upstream STING signal molecule cGAS. We also did not observe any difference in the frequency of proliferating GC B cells in the STING-Gt *vs.* WT compartment of STING-Gt mixed chimeras, suggesting that STING signaling did not regulate cell death signaling in our model. However, human immune cells have been reported to be particularly sensitive to STING mediated cell death, indicating that there could be important species differences in this aspect of the pathway (45). Although the STING-Gt model has been verified in many independent studies as a robust model of STING deficiency, we noted that previous reports of STING functions in B cells were based upon complete or conditional knock-out of STING. We verified that the STING-Gt mutation faithfully recapitulated a complete block in STING signaling.

The simplest explanation why we, in contrast to the diverging results of previous reports, do not observe any role of STING may be the nature of the response itself. Whereas the prior studies were focused on foreign antigen challenge by immunization, we investigated chronic, autoreactive GC responses. Of note, although we did not see an impact on PC formation in the COI of the STING-Gt B cell model, we did observe a significant, albeit small, effect on PC levels deriving from the COI of the cGAS KO model. Although this seems to be in line with the results of Walker and colleagues, it could also be a secondary effect of PC survival signals driven through this pathway, as was previously suggested for TLR5 in vaccination settings (48). We cannot definitively exclude the possibility that STING deficiency influences autoantibody production in our model. Because only half of cells derive from STING-Gt or cGAS KO cells, respectively in the two models, we cannot reliably assess Ab production deriving, specifically, from these cells in the intact animal.

Our findings are overall in line with a recent study reporting that the cGAS-STING pathway does not promote autoimmunity in two murine models of SLE, the pristane induced lupus model and the genetically programmed MRL/lpr model (49). Whereas that study investigated the role of global cGAS or STING knock-out, thus affecting myeloid, lymphoid and stromal cells alike, the present study interrogated the role of the cGAS-STING pathway in GC B cells specifically. Whereas potential counteractive effects are possible in the complex scenario of global knock-out of cGAS and STING in the study by Motwani et al., our observations demonstrate that B cell intrinsic cGAS-STING signaling does not influence autoreactive GC B cell responses. In the context of potential STING activity in human autoreactive B cells and human autoimmune diseases, however, the threshold for cGAS/STING pathway activation and type I interferon secretion could be different. Further studies are needed to fully elucidate such potential species differences.

One of the main downstream products of STING activation is Type I IFN, which acts on IFNAR. Walker and colleagues suggested that the effects of STING they observed in part depended on B cell intrinsic IFNAR signaling. However, the B cell intrinsic importance of IFNAR has also been debated. Knock-out of IFNAR in a Wiskott-Aldrich syndrome (WAS) chimera model of B cell-driven autoimmunity, where B cell-specific WAS protein deficiency results in hyperresponsive B cells, did not block development of humoral autoimmunity, nor did it prevent the generation of robust splenic GCs (28). Conversely, two recent studies investigating the influence of B cell specific IFNAR KO in spontaneous SLE settings (B6.Sle1b IFNAR^-/-^: µMT chimeras or B6.Nba2 Mb1-Cre IFNAR^flx/flx^ mice) found a decreased GCB cell population in mice with IFNAR deficient B cells (25, 50). In line with the findings from the WAS model, we did not see any impact of a block in para-autocrine signaling through IFNAR in our model. The discrepancy from the results of Keller and colleagues may be explained by the partial *vs.* complete absence of IFNAR in B cells. In this context, it is at the same time a strength and a weakness of our model that we have internally competing B cell populations. We can rather definitively make conclusions on the B cell intrinsic roles of the investigated signaling components, but we cannot account for potential secondary signals or compensatory effects, that could be mediated by the interplay with a sizeable WT B cell population. The difference in sensitivity of our model to IFNAR blockade may, in turn, partly explain the observed difference in sensitivity to STING blockade.

In contrast to the lack of impact of cGAS, STING, or IFNAR deficiency, our findings on the near-absolute importance of TLR7 in GC B cell responses is well-aligned with numerous prior studies (15, 51–53). TLR7 and STING signal transduction both induce type I IFN expression, although the former does so through IRF-7 and the latter through IRF-3. Through knock-out of IFNAR, we found the B cell intrinsic action of type I IFN to be expendable for their GC participation. In addition, the two signaling pathways converge, albeit ostensibly *via* different routes, on the activation of MAPK/AP-1 and NF-κB, which promote production of proinflammatory cytokines, survival, and proliferation of the cell (54). Despite their downstream overlap in effector functions, the TLR7 and STING pathways are compartmentalized very differently in the host cell, with topological equivalence and inequivalence, respectively, to the subcellular localization of the BCR. Taken together, our findings are in line with the notion that physiological B cell responses are strictly supported by signals linked to BCR-mediated endocytosis, as previously demonstrated for dual-specific antigens which carry TLR7 or TLR9 ligands. In fact, it has been found that signaling through the endosomally located TLR7 can directly cooperate and cross-talk with aberrant BCR signals (55). Conversely, intracellular signals that could arise from, *e.g.*, viral infection of B cells are precluded from short-circuiting B cell activities. We speculate that this wiring of B cell signals is essential to enable appropriate antibody responses, while at the same time restricting aberrant antibody responses during infections and in autoimmune or autoinflammatory settings. The notion that B cell intrinsic STING signals do not support autoimmune progression in and of themselves, provides some reassurance that anti-cancer therapies relying on STING agonists, which are currently in development (56), will not pose a significant direct risk of breaking B cell tolerance.

## Data Availability Statement

The raw data supporting the conclusions of this article will be made available by the authors, without undue reservation.

## Ethics Statement

The animal study was reviewed and approved by The Danish Animal Experiments Inspectorate.

## Author Contributions

SD conceived and designed the study. KG, TW, and SD planned and designed experiments. KG, TW, CF-H, ET-D, NvC, and LJ carried out experiments. LR, RH, and SP provided essential reagents and tools, and provided valuable feedback on experimental design. KG, TW, and SD analysed the data and made figures. SD wrote the first draft of the manuscript. All authors contributed to the article and approved the submitted version.

## Funding

This work was supported by the Novo Nordisk Foundation (NNF17OC0028160) and the Independent Research Fund Denmark through a Sapere Aude Research Leader grant to SD (9060-00038B). The SP laboratory was supported by grants from the Novo Nordisk Foundation (NNF20OC0063436) and The European Research Council (786602). CF-H was supported by a postdoctoral fellowship from Lundbeckfonden (R303-2018-3415).

## Conflict of Interest

The authors declare that the research was conducted in the absence of any commercial or financial relationships that could be construed as a potential conflict of interest.

## Publisher’s Note

All claims expressed in this article are solely those of the authors and do not necessarily represent those of their affiliated organizations, or those of the publisher, the editors and the reviewers. Any product that may be evaluated in this article, or claim that may be made by its manufacturer, is not guaranteed or endorsed by the publisher.

## Supplementary Material

Supplementary Figure 1 Assessment of chimerism for the IFNAR mixed BM chimeras and controls. **(A)** B cell frequencies out of live, singlet lymphocytes, in blood of IFNAR-KO (blue, n = 6) and WT (red, n = 5) chimeras. Each dot represents an individual mouse and bars indicate mean +/- SD, with statistical significance given for unpaired t test (α = 0.05), ns = not significant. **(B)** As in **(A)**, but for CD4^+^ T cell frequencies instead. **(C)** As in **(A)**, but for CD8^+^ T cell frequencies instead. **(D)** CD45.1 *vs.* CD45.2 stratification of the CD4^+^ T cells from **(B)**. Each dot represents an individual mouse and bars indicate mean +/- SD, dotted lines illustrate expected frequencies of 33.3% and 66.6% on the y-axis. **(E)** As in **(D)**, but for the CD8^+^ T cells from **(C)** instead. **(F)** as for **(D)**, but for B cells from **(A)** instead, only stratifying for CD45.2 expression and with dotted line at expected frequency of 50% on the y-axis. **(G)** As **(A)**, but for 564Igi idiotype (9D11^+^) frequencies of B cells. **(H)** Anti-dsDNA Ab in blood of IFNAR-KO (blue, n = 6) and WT (red, n = 5) chimeras. Each dot represents an individual mouse and bars indicate mean +/- SD, with statistical significance given for unpaired t test (α = 0.05), ns = not significant. Grayed area represents the background level of C57BL6 mice. **(I)** Relative Cxcl10/β-actin mRNA expression in bone marrow-derived IFNAR-KO (blue) or WT (red) macrophages, upon mock stimulation or stimulation with IFN-β, as indicated. Each dot represents an experimental replicate, and bars indicate mean +/- SD, with statistical significance given for two-way ANOVA followed by Sidak’s post-test (α = 0.05), ns = not significant, **** = p<0.0001. **(J)** Confocal micrograph of spleen section from a representative IFNAR-KO chimera, stained for CD169 (green) for marginal zone indication and IgD (red) to indicate follicles and GCs (exclusion zones). **(K)** IFNAR positive cell percentages within the CD45.1 and CD45.2 B cell compartments in IngLN of IFNAR-KO and WT control chimeras. Each dot represents a mouse, and bars indicate median +/- SD, with statistical significance given for two-way ANOVA followed by Sidak’s post-test (α = 0.05), ns = not significant, **** = p<0.0001. **(L)** as in J, but for mesLN. **(M)** as in J, but for spleen. **(N)** as in J, but for the GCB compartment. **(O)** as in K, but for the GCB compartment. **(P)** as in L, but for the GCB compartment.

Supplementary Figure 2 Assessment of chimerism for the STING mixed BM chimeras and controls presented in **Figure 2**, **(A)** B cell frequencies out of live, singlet lymphocytes, in blood of STING-Gt (blue, n = 5) and WT (red, n = 5) chimeras. Each dot represents an individual mouse and bars indicate mean +/- SD, with statistical significance given for unpaired t test (alpha = 0.05), ns = not significant. **(B)** As in **(A)**, but for T cell frequencies instead. **(C)** CD45.1 *vs.* CD45.2 stratification of the T cells from **(B)**. Each dot represents an individual mouse and bars indicate mean +/- SD, dotted lines illustrate expected frequencies of 33.3% and 66.6% on the y-axis. **(D)** as for **(C)**, but for B cells from **(A)** instead, only stratifying for CD45.2 expression and with dotted line at expected frequency of 50% on the y-axis. **(E)** As **(A)**, but for 564Igi idiotype (9D11^+^) frequencies of B cells. **(F)** Anti-dsDNA Ab in blood of STING-Gt (blue, n = 5) and WT (red, n = 5) chimeras. Each dot represents an individual mouse and bars indicate mean +/- SD, with statistical significance given for unpaired t test (alpha = 0.05), ns = not significant. Grayed area represents the background level of C57BL6 mice. **(G)** Confocal micrograph of spleen section from a representative STING-Gt chimera, stained for CD169 (green) for marginal zone indication and IgD (red) to indicate follicles and GCs (exclusion zones).

Supplementary Figure 3 Assessment of chimerism for the STING mixed BM chimeras and controls presented in **Figure 3**, and impact of the STING Golden-ticket mutation on protein expression and enzymatic activity. **(A)** B cell frequencies out of live, singlet lymphocytes, in blood of STING-Gt (blue, n = 6) and WT (red, n = 7) chimeras. Each dot represents an individual mouse and bars indicate mean +/- SD, with statistical significance given for unpaired t test (alpha = 0.05), ns = not significant. **(B)** As in **(A)**, but for CD4^+^ T cell frequencies instead. **(C)** As in **(A)**, but for CD8^+^ T cell frequencies instead. **(D)** CD45.1^neg^ fraction of CD4^+^ T cells from **(B)**, stratified for PA-GFP status. Black bars indicate mean of CD45.1 PA-GFP double negative cells, whereas pink bars indicate mean of CD45.1 negative PA-GFP positive cells. Error bars indicate the SD of the mean. Dotted lines illustrate expected frequencies of 33.3% and 66.6% on the y-axis. **(E)** As in **(D)**, but for CD8^+^ T cells from **(C)** instead. **(F)** As in **(D)** and **(E)** but illustrating CD45.1 positive compartments of the CD4^+^ and CD8^+^ T cells, respectively. Dotted line illustrates the expected frequency of 33.3% on the y-axis. **(G)** as for **(D)**, but for B cells from **(A)** instead, and with dotted line at the expected frequency of 50% on the y-axis. **(H)** As **(A)**, but for 564Igi idiotype (9D11^+^) frequencies of B cells. **(I)** Anti-dsDNA Ab in blood of STING-Gt (blue, n = 6) and WT (red, n = 7) chimeras. Each dot represents an individual mouse and bars indicate mean +/- SD, with statistical significance given for unpaired t test (alpha = 0.05), ns = not significant. Grayed area represents the background level of C57BL6 mice. **(J)** Two confocal micrographs of spleen sections from a representative STING-Gt chimera, stained for CD169 (green) for marginal zone indication and IgD (red) to indicate follicles and GCs (exclusion zones). **(K)** Western blot of FLAG-tagged WT STING (WT) and STING-Gt (I199N) both with an expected MW of ~44 kDa. GFP transfected cells served as a negative control. The blot was developed with anti-FLAG mAb. **(L)** Western blot of FLAG-tagged WT STING (WT) and STING-Gt (I199N) both with an expected MW of ~44 kDa. GFP transfected cells served as a negative control. The blot was developed with anti-STING mAb. **(M)** STING activity assay utilizing Firefly and Renilla luciferases to measure downstream enzymatic activity in lysates of STING-Gt, WT or GFP control cells. Each dot represents an experimental replicate, and bars indicate mean +/- SD, with statistical significance given for two-way ANOVA followed by Sidak’s post-test (alpha = 0.05), ns = not significant, ** = p<0.01. **(N)** Relative IFN-β/β-actin mRNA expression in bone marrow-derived STING-Gt (blue) or WT (red) macrophages, upon mock stimulation or stimulation with dsDNA, as indicated. Each dot represents an experimental replicate, and bars indicate mean +/- SD, with statistical significance given for two-way ANOVA followed by Sidak’s post-test (alpha = 0.05), ns = not significant, **** = p<0.0001.

Supplementary Figure 4 Assessment of chimerism for the TLR7 mixed BM chimeras and controls. **(A)** B cell frequencies out of live, singlet lymphocytes, in blood of TLR7-KO (blue, n = 5) and WT (red, n = 5) chimeras. Each dot represents an individual mouse and bars indicate mean +/- SD, with statistical significance given for unpaired t test (α = 0.05), ns = not significant. **(B)** As in **(A)**, but for CD4^+^ T cell frequencies instead. **(C)** As in **(A)**, but for CD8^+^ T cell frequencies instead. **(D)** CD45.1^pos^ fraction of CD4^+^ T cells from **(B)**, stratified for PA-GFP status. Black bars indicate mean of CD45.1 positive PA-GFP negative cells, whereas pink bars indicate mean of CD45.1 PA-GFP double positive cells. Error bars indicate the SD of the mean. Dotted lines illustrate expected frequencies of 33.3% and 66.6% on the y-axis. **(E)** As in **(D)**, but for CD8^+^ T cells from **(C)** instead. **(F)** As in **(D)** and **(E)** but illustrating CD45.1 negative compartments of the CD4^+^ and CD8^+^ T cells, respectively. Dotted line illustrates the expected frequency of 33.3% on the y-axis. **(G)** as for **(D)**, but for B cells from **(A)** instead, and with dotted line at the expected frequency of 50% on the y-axis. **(H)** As **(A)**, but for 564Igi idiotype (9D11^+^) frequencies of B cells. **(I)** Anti-dsDNA Ab in blood of TLR7-KO (blue, n = 5) and WT (red, n = 5) chimeras. Each dot represents an individual mouse and bars indicate mean +/- SD, with statistical significance given for unpaired t test (α = 0.05), ns = not significant. Grayed area represents the background level of C57BL6 mice. **(J)** Confocal micrograph of spleen section from a representative TLR7-KO chimera (left) and a WT control (right), stained for CD169 (green) for marginal zone indication and IgD (red) to indicate follicles and GCs (exclusion zones).

Supplementary Figure 5 Assessment of chimerism for the cGAS mixed BM chimeras and controls. **(A)** B cell frequencies out of live, singlet lymphocytes, in blood of cGAS-KO (blue, n = 7) and WT (red, n = 7) chimeras. Each dot represents an individual mouse and bars indicate mean +/- SD, with statistical significance given for unpaired t test (α = 0.05), ns = not significant, * = p<0.05. **(B)** As in **(A)**, but for CD4^+^ T cell frequencies instead. **(C)** As in **(A)**, but for CD8^+^ T cell frequencies instead. **(D)** CD45.1^neg^ fraction of CD4^+^ T cells from **(B)**, stratified for PA-GFP status. Black bars indicate mean of CD45.1 PA-GFP double negative cells, whereas pink bars indicate mean of CD45.1 negative PA-GFP positive cells. Error bars indicate the SD of the mean. Dotted lines illustrate expected frequencies of 33.3% and 66.6% on the y-axis. **(E)** As in **(D)**, but for CD8^+^ T cells from **(C)** instead. **(F)** As in **(D)** and **(E)** but illustrating CD45.1 positive compartments of the CD4^+^ and CD8^+^ T cells, respectively. Dotted line illustrates the expected frequency of 33.3% on the y-axis. **(G)** as **(D)**, but for B cells from **(A)** instead and with dotted line at the expected frequency of 50% on the y-axis. **(H)** As **(A)**, but for 564Igi idiotype (9D11^+^) frequencies of B cells. **(I)** Anti-dsDNA Ab in blood of cGAS-KO (blue, n = 7) and WT (red, n = 7) chimeras. Each dot represents an individual mouse and bars indicate mean +/- SD, with statistical significance given for unpaired t-test (α = 0.05), ns = not significant, ** = p<0.01. Grayed area represents the background level of C57BL6 mice. **(J)** Relative IFN-β/β-actin mRNA expression in bone marrow-derived cGAS-KO (blue) or WT (red) macrophages, upon mock stimulation or stimulation with dsDNA, as indicated. Each dot represents an experimental replicate, and bars indicate mean +/- SD, with statistical significance given for two-way ANOVA followed by Sidak’s post-test (α = 0.05), ns = not significant, **** = p<0.0001. **(K)** Confocal micrograph of spleen section from a representative cGAS-KO chimera, stained for CD169 (green) for marginal zone indication and IgD (red) to indicate follicles and GCs (exclusion zones).
