# Supplementary figures and images for: B Cell Intrinsic STING Signaling Is Not Required for Autoreactive Germinal Center Participation
Source: Front Immunol. 2021 Dec 6;12:782558. doi: 10.3389/fimmu.2021.782558 (PMC8685402; doi:10.3389/fimmu.2021.782558)

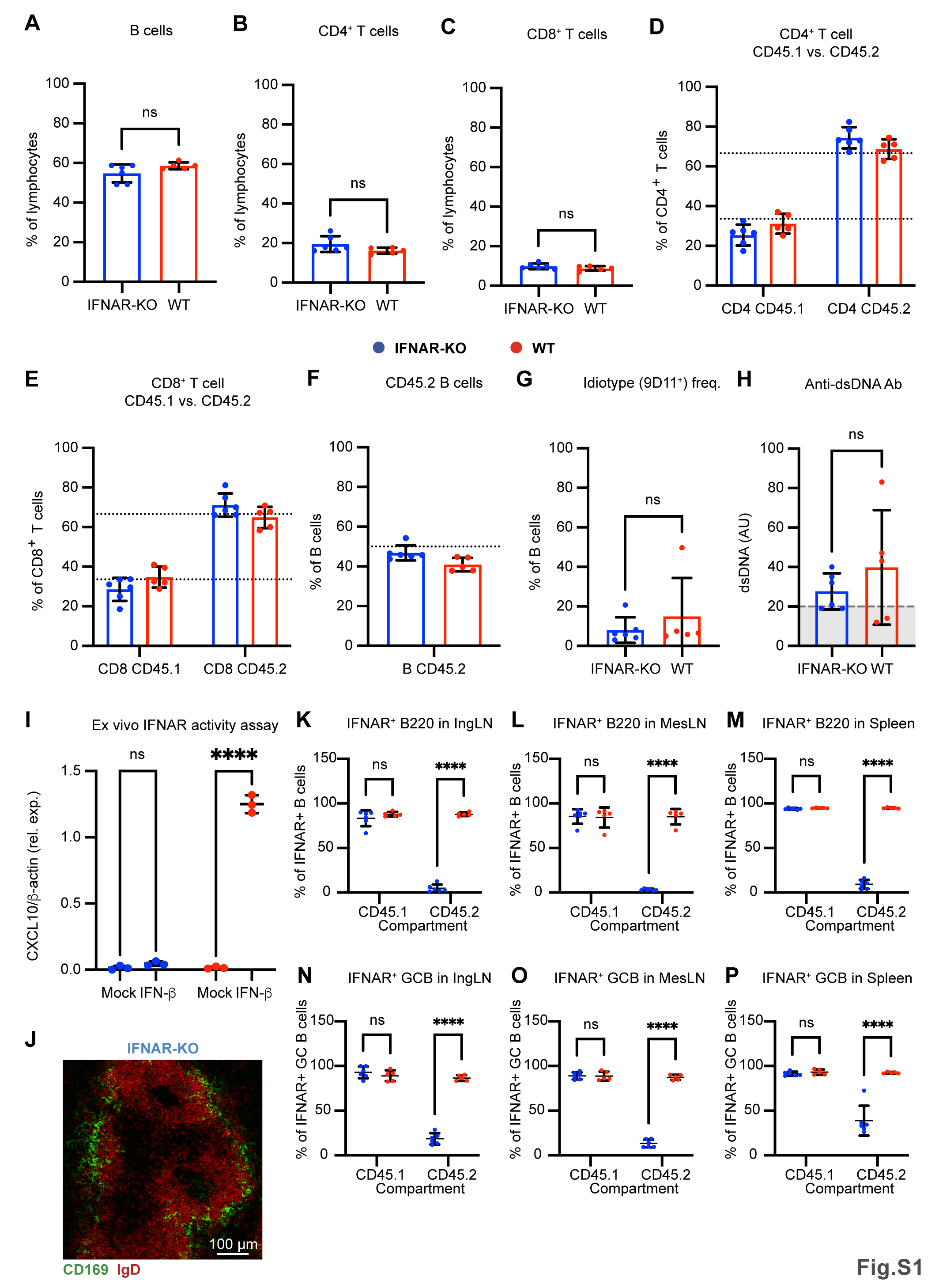

Supplement: Supplementary Figure 1 — Assessment of chimerism for the IFNAR mixed BM chimeras and controls. (A) B cell frequencies out of live, singlet lymphocytes, in blood of IFNAR-KO (blue, n = 6) and WT (red, n = 5) chimeras. Each dot represents an individual mouse and bars indicate mean +/- SD, with statistical significance given for unpaired t test (α = 0.05), ns = not significant. (B) As in (A), but for CD4+ T cell frequencies instead. (C) As in (A), but for CD8+ T cell frequencies instead. (D) CD45.1 vs. CD45.2 stratification of the CD4+ T cells from (B). Each dot represents an individual mouse and bars indicate mean +/- SD, dotted lines illustrate expected frequencies of 33.3% and 66.6% on the y-axis. (E) As in (D), but for the CD8+ T cells from (C) instead. (F) as for (D), but for B cells from (A) instead, only stratifying for CD45.2 expression and with dotted line at expected frequency of 50% on the y-axis. (G) As (A), but for 564Igi idiotype (9D11+) frequencies of B cells. (H) Anti-dsDNA Ab in blood of IFNAR-KO (blue, n = 6) and WT (red, n = 5) chimeras. Each dot represents an individual mouse and bars indicate mean +/- SD, with statistical significance given for unpaired t test (α = 0.05), ns = not significant. Grayed area represents the background level of C57BL6 mice. (I) Relative Cxcl10/β-actin mRNA expression in bone marrow-derived IFNAR-KO (blue) or WT (red) macrophages, upon mock stimulation or stimulation with IFN-β, as indicated. Each dot represents an experimental replicate, and bars indicate mean +/- SD, with statistical significance given for two-way ANOVA followed by Sidak’s post-test (α = 0.05), ns = not significant, **** = p<0.0001. (J) Confocal micrograph of spleen section from a representative IFNAR-KO chimera, stained for CD169 (green) for marginal zone indication and IgD (red) to indicate follicles and GCs (exclusion zones). (K) IFNAR positive cell percentages within the CD45.1 and CD45.2 B cell compartments in IngLN of IFNAR-KO and WT control chimeras. Each dot represents [file Image_1.tif]

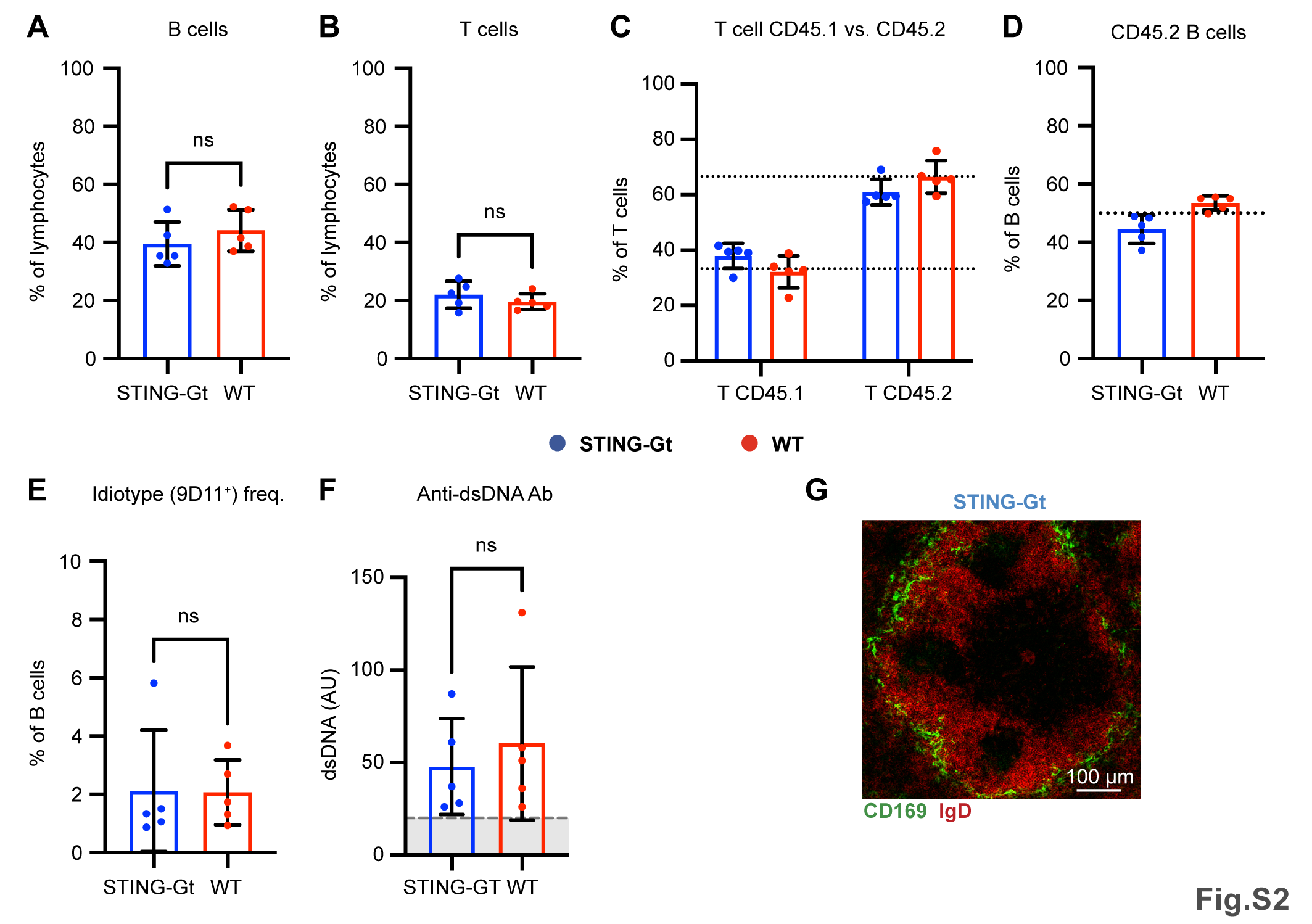

Supplement: Supplementary Figure 2 — Assessment of chimerism for the STING mixed BM chimeras and controls presented in Figure 2 , (A) B cell frequencies out of live, singlet lymphocytes, in blood of STING-Gt (blue, n = 5) and WT (red, n = 5) chimeras. Each dot represents an individual mouse and bars indicate mean +/- SD, with statistical significance given for unpaired t test (alpha = 0.05), ns = not significant. (B) As in (A), but for T cell frequencies instead. (C) CD45.1 vs. CD45.2 stratification of the T cells from (B). Each dot represents an individual mouse and bars indicate mean +/- SD, dotted lines illustrate expected frequencies of 33.3% and 66.6% on the y-axis. (D) as for (C), but for B cells from (A) instead, only stratifying for CD45.2 expression and with dotted line at expected frequency of 50% on the y-axis. (E) As (A), but for 564Igi idiotype (9D11+) frequencies of B cells. (F) Anti-dsDNA Ab in blood of STING-Gt (blue, n = 5) and WT (red, n = 5) chimeras. Each dot represents an individual mouse and bars indicate mean +/- SD, with statistical significance given for unpaired t test (alpha = 0.05), ns = not significant. Grayed area represents the background level of C57BL6 mice. (G) Confocal micrograph of spleen section from a representative STING-Gt chimera, stained for CD169 (green) for marginal zone indication and IgD (red) to indicate follicles and GCs (exclusion zones). [file Image_2.tif]

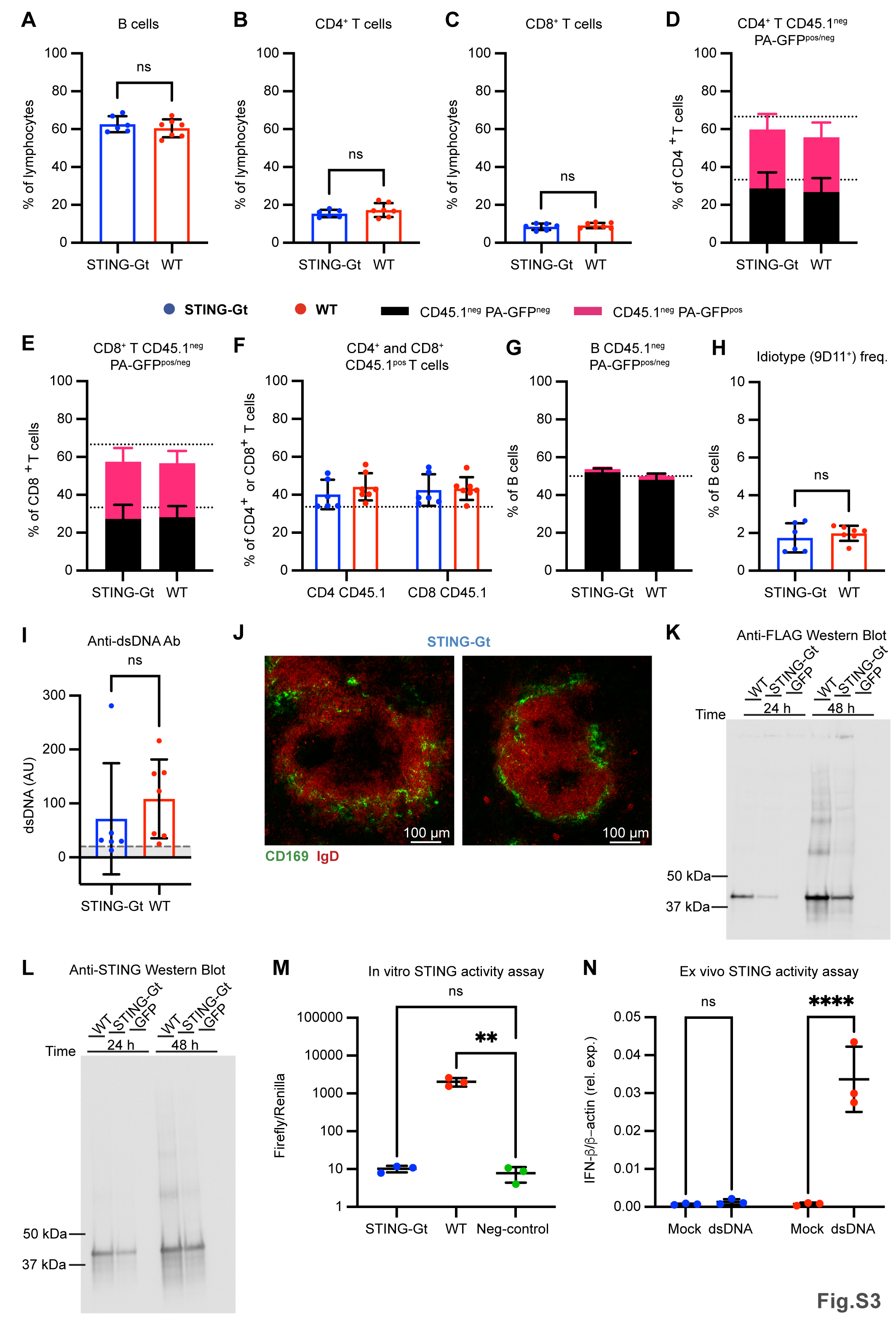

Supplement: Supplementary Figure 3 — Assessment of chimerism for the STING mixed BM chimeras and controls presented in Figure 3 , and impact of the STING Golden-ticket mutation on protein expression and enzymatic activity. (A) B cell frequencies out of live, singlet lymphocytes, in blood of STING-Gt (blue, n = 6) and WT (red, n = 7) chimeras. Each dot represents an individual mouse and bars indicate mean +/- SD, with statistical significance given for unpaired t test (alpha = 0.05), ns = not significant. (B) As in (A), but for CD4+ T cell frequencies instead. (C) As in (A), but for CD8+ T cell frequencies instead. (D) CD45.1neg fraction of CD4+ T cells from (B), stratified for PA-GFP status. Black bars indicate mean of CD45.1 PA-GFP double negative cells, whereas pink bars indicate mean of CD45.1 negative PA-GFP positive cells. Error bars indicate the SD of the mean. Dotted lines illustrate expected frequencies of 33.3% and 66.6% on the y-axis. (E) As in (D), but for CD8+ T cells from (C) instead. (F) As in (D) and (E) but illustrating CD45.1 positive compartments of the CD4+ and CD8+ T cells, respectively. Dotted line illustrates the expected frequency of 33.3% on the y-axis. (G) as for (D), but for B cells from (A) instead, and with dotted line at the expected frequency of 50% on the y-axis. (H) As (A), but for 564Igi idiotype (9D11+) frequencies of B cells. (I) Anti-dsDNA Ab in blood of STING-Gt (blue, n = 6) and WT (red, n = 7) chimeras. Each dot represents an individual mouse and bars indicate mean +/- SD, with statistical significance given for unpaired t test (alpha = 0.05), ns = not significant. Grayed area represents the background level of C57BL6 mice. (J) Two confocal micrographs of spleen sections from a representative STING-Gt chimera, stained for CD169 (green) for marginal zone indication and IgD (red) to indicate follicles and GCs (exclusion zones). (K) Western blot of FLAG-tagged WT STING (WT) and STING-Gt (I199N) both with an expected MW of ~44 kDa. GFP transfected cells served as a ne [file Image_3.tif]

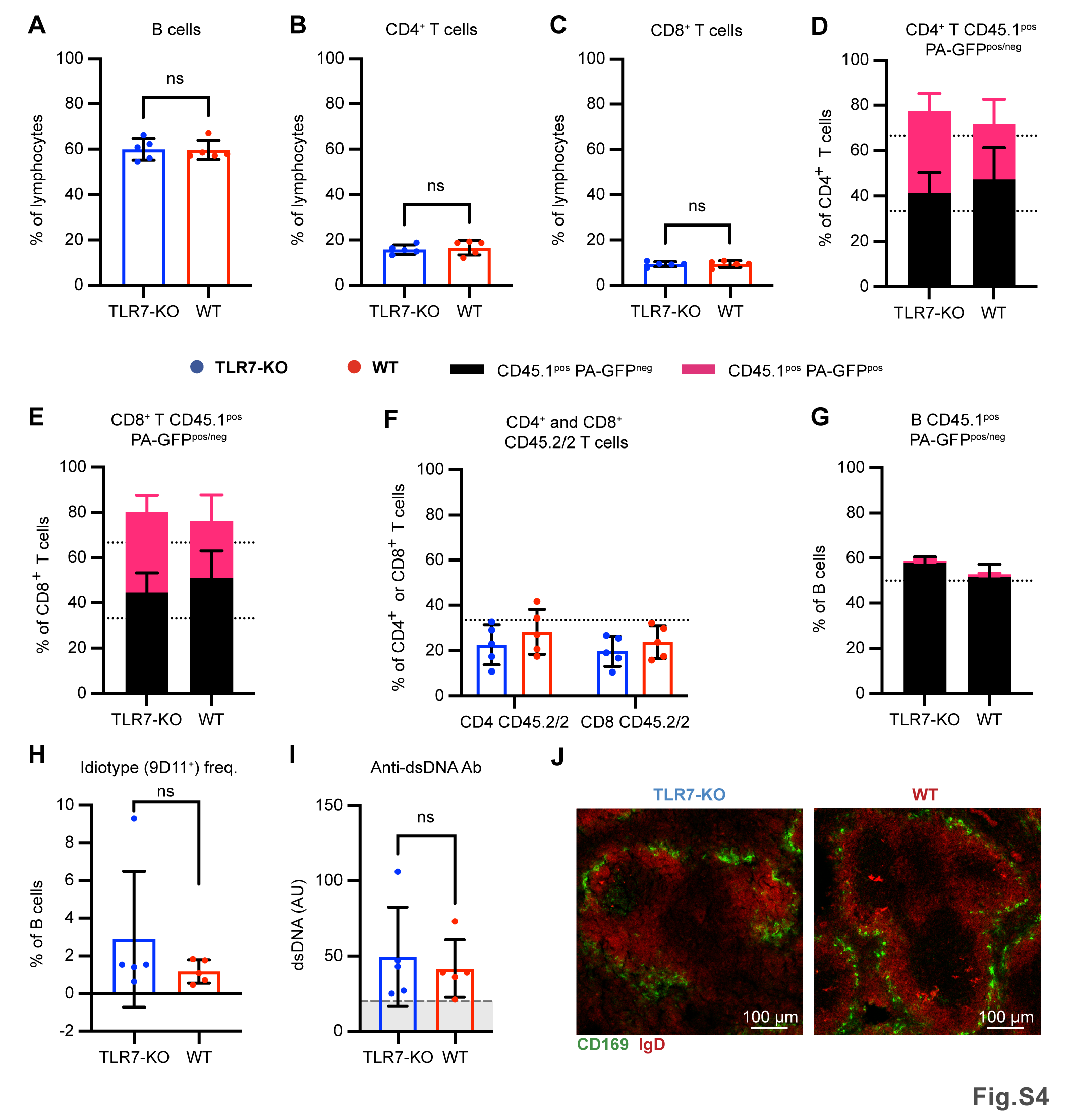

Supplement: Supplementary Figure 4 — Assessment of chimerism for the TLR7 mixed BM chimeras and controls. (A) B cell frequencies out of live, singlet lymphocytes, in blood of TLR7-KO (blue, n = 5) and WT (red, n = 5) chimeras. Each dot represents an individual mouse and bars indicate mean +/- SD, with statistical significance given for unpaired t test (α = 0.05), ns = not significant. (B) As in (A), but for CD4+ T cell frequencies instead. (C) As in (A), but for CD8+ T cell frequencies instead. (D) CD45.1pos fraction of CD4+ T cells from (B), stratified for PA-GFP status. Black bars indicate mean of CD45.1 positive PA-GFP negative cells, whereas pink bars indicate mean of CD45.1 PA-GFP double positive cells. Error bars indicate the SD of the mean. Dotted lines illustrate expected frequencies of 33.3% and 66.6% on the y-axis. (E) As in (D), but for CD8+ T cells from (C) instead. (F) As in (D) and (E) but illustrating CD45.1 negative compartments of the CD4+ and CD8+ T cells, respectively. Dotted line illustrates the expected frequency of 33.3% on the y-axis. (G) as for (D), but for B cells from (A) instead, and with dotted line at the expected frequency of 50% on the y-axis. (H) As (A), but for 564Igi idiotype (9D11+) frequencies of B cells. (I) Anti-dsDNA Ab in blood of TLR7-KO (blue, n = 5) and WT (red, n = 5) chimeras. Each dot represents an individual mouse and bars indicate mean +/- SD, with statistical significance given for unpaired t test (α = 0.05), ns = not significant. Grayed area represents the background level of C57BL6 mice. (J) Confocal micrograph of spleen section from a representative TLR7-KO chimera (left) and a WT control (right), stained for CD169 (green) for marginal zone indication and IgD (red) to indicate follicles and GCs (exclusion zones). [file Image_4.tif]

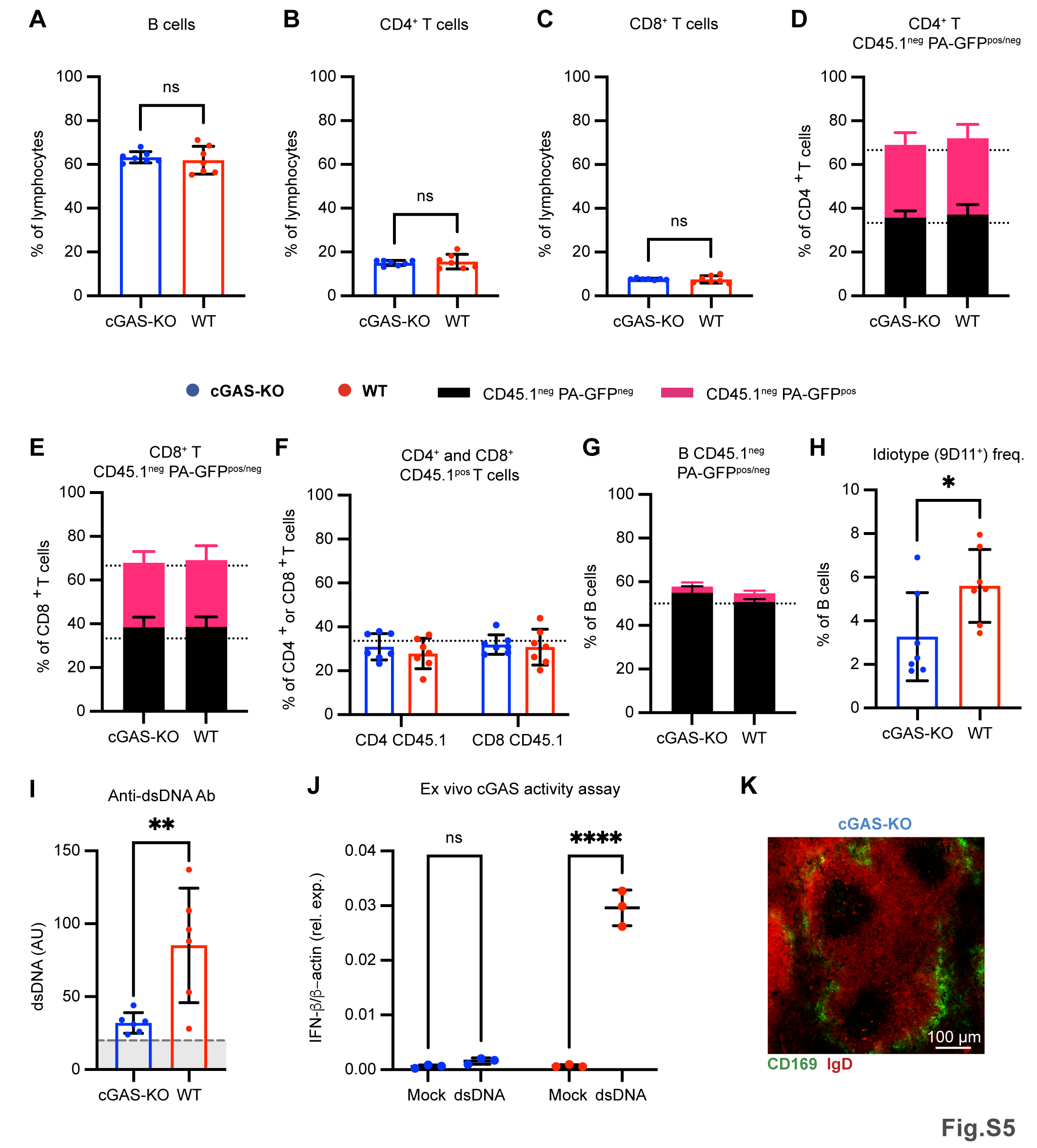

Supplement: Supplementary Figure 5 — Assessment of chimerism for the cGAS mixed BM chimeras and controls. (A) B cell frequencies out of live, singlet lymphocytes, in blood of cGAS-KO (blue, n = 7) and WT (red, n = 7) chimeras. Each dot represents an individual mouse and bars indicate mean +/- SD, with statistical significance given for unpaired t test (α = 0.05), ns = not significant, * = p<0.05. (B) As in (A), but for CD4+ T cell frequencies instead. (C) As in (A), but for CD8+ T cell frequencies instead. (D) CD45.1neg fraction of CD4+ T cells from (B), stratified for PA-GFP status. Black bars indicate mean of CD45.1 PA-GFP double negative cells, whereas pink bars indicate mean of CD45.1 negative PA-GFP positive cells. Error bars indicate the SD of the mean. Dotted lines illustrate expected frequencies of 33.3% and 66.6% on the y-axis. (E) As in (D), but for CD8+ T cells from (C) instead. (F) As in (D) and (E) but illustrating CD45.1 positive compartments of the CD4+ and CD8+ T cells, respectively. Dotted line illustrates the expected frequency of 33.3% on the y-axis. (G) as (D), but for B cells from (A) instead and with dotted line at the expected frequency of 50% on the y-axis. (H) As (A), but for 564Igi idiotype (9D11+) frequencies of B cells. (I) Anti-dsDNA Ab in blood of cGAS-KO (blue, n = 7) and WT (red, n = 7) chimeras. Each dot represents an individual mouse and bars indicate mean +/- SD, with statistical significance given for unpaired t-test (α = 0.05), ns = not significant, ** = p<0.01. Grayed area represents the background level of C57BL6 mice. (J) Relative IFN-β/β-actin mRNA expression in bone marrow-derived cGAS-KO (blue) or WT (red) macrophages, upon mock stimulation or stimulation with dsDNA, as indicated. Each dot represents an experimental replicate, and bars indicate mean +/- SD, with statistical significance given for two-way ANOVA followed by Sidak’s post-test (α = 0.05), ns = not significant, **** = p<0.0001. (K) Confocal micrograph of spleen section from a representative cGAS-KO [file Image_5.tif]
